# Multilevel thresholding with divergence measure and improved particle swarm optimization algorithm for crack image segmentation

**DOI:** 10.1038/s41598-024-58456-2

**Published:** 2024-04-01

**Authors:** Fangyan Nie, Mengzhu Liu, Pingfeng Zhang

**Affiliations:** 1 Computer and Information Engineering College, Guizhou University of Commerce, Guiyang, 550014 China; 2 College of Marxism, Guizhou University of Commerce, Guiyang, 550014 China

**Keywords:** Crack detection, Multilevel image thresholding, Minimum arithmetic-geometric divergenc, Particle swarm optimization, Local stochastic perturbation, Computer science, Information technology, Electrical and electronic engineering

## Abstract

Crack formation is a common phenomenon in engineering structures, which can cause serious damage to the safety and health of these structures. An important method of ensuring the safety and health of engineered structures is the prompt detection of cracks. Image threshold segmentation based on machine vision is a crucial technology for crack detection. Threshold segmentation can separate the crack area from the background, providing convenience for more accurate measurement and evaluation of the crack condition and location. The segmentation of cracks in complex scenes is a challenging task, and this goal can be achieved by means of multilevel thresholding. The arithmetic-geometric divergence combines the advantages of the arithmetic mean and the geometric mean in probability measures, enabling a more precise capture of the local features of an image in image processing. In this paper, a multilevel thresholding method for crack image segmentation based on the minimum arithmetic-geometric divergence is proposed. To address the issue of time complexity in multilevel thresholding, an enhanced particle swarm optimization algorithm with local stochastic perturbation is proposed. In crack detection, the thresholding criterion function based on the minimum arithmetic-geometric divergence can adaptively determine the thresholds according to the distribution characteristics of pixel values in the image. The proposed enhanced particle swarm optimization algorithm can increase the diversity of candidate solutions and enhance the global convergence performance of the algorithm. The proposed method for crack image segmentation is compared with seven state-of-the-art multilevel thresholding methods based on several metrics, including RMSE, PSNR, SSIM, FSIM, and computation time. The experimental results show that the proposed method outperforms several competing methods in terms of these metrics.

## Introduction

Cracks pose a significant risk to the safety and health of structures such as bridges, dams, pavements, walls, and tunnels. If not detected promptly, they can cause irreversible and extensive damage^1^. Therefore, in the construction and maintenance of modern buildings, crack detection plays a crucial role^2^. Due to their efficiency and capability to operate in areas that are inaccessible (or extremely dangerous) to humans, machine vision-based methods (e.g., crack detection using unmanned aerial vehicles) have become one of the most commonly utilized techniques for crack detection^3,4^. Among machine vision-based crack detection methods, image segmentation techniques based on image thresholding are widely used due to their ease of implementation and superior real-time performance^5–7^.

Image thresholding has numerous applications in contemporary social life and industrial operations^8–10^. Because of its ease of implementation, high efficiency, and excellent real-time performance, it has caught the interest of many researchers and is widely utilized in engineering. However, there are also considerable obstacles in crack detection with thresholding in practical circumstances. For instance, when the image background is complex or the lighting conditions are poor or uneven, crack detection based on image thresholding often fails to yield satisfactory results. Multilevel thresholding is an effective approach to improve the segmentation performance of thresholding. In many cases, when a single threshold does not provide satisfactory segmentation results for certain scenarios, multiple thresholds can be set to achieve the segmentation goals. Therefore, numerous studies have developed various multilevel thresholding methods to enhance image segmentation performance and enable practical applications. For example, multilevel thresholding has been widely and successfully applied in areas such as medical diagnosis^11,12^, power equipment fault detection^13^, and crop image segmentation^14^.

The segmentation performance is improved by multilevel thresholding. However, the time complexity of the algorithm has also increased sharply. As the number of thresholds increases, the computation time also increases exponentially. Many studies have incorporated swarm intelligence optimization algorithms into image multilevel thresholding problems to solve this problem^15–20^. Segmentation is a multi-constraint optimization problem when using swarm intelligence optimization algorithms for multilevel thresholding. In general, multilevel thresholding is formulated as a maximum or minimum solution problem. Under constrained conditions, the problem will be solved through several rounds of iteration. In this scheme, the problem model is summarized as an extremum problem based on a specific criterion function. In practical applications, the acquisition of the final image segmentation results is also strongly influenced by the choice of the criterion functions. The commonly used criterion functions include the Otsu criterion^21,22^ based on the maximum inter-class variance, the maximum Shannon entropy criterion^23,24^, and the minimum cross entropy criterion^14,25^, etc. For the Otsu criterion, the maximum Shannon entropy criterion, and the minimum cross entropy criterion, they have similar properties. Namely, when the gray level distribution of the image closely resembles a uniform distribution, these criteria yield the best results. In the real environment, there is a significant difference in the gray level distribution between image targets and backgrounds. This disparity highlights the limitations of these criteria in image segmentation. In many cases, the optimal segmentation threshold tends to be on the side where the gray level distribution dominates.

Based on the study of the traditional divergence theory of information theory, Taneja proposes a new information theory measure, namely the arithmetic and geometric divergence measure^26^, which addresses the limitations of the traditional information divergence in quantifying the similarity or dissimilarity between distinct probability distribution systems. As an information theory distance measure, arithmetic and geometric divergence measures can effectively quantify the differences between information systems. Image is a typical information system. Segmenting an image involves creating a segmented image that accurately represents the original image, effectively distinguishing between the background and the target of the original image. From the perspective of information theory, it is important to preserve as much of the original image information as possible when segmenting the image. Since the arithmetic and geometric divergence measure provides an information metric criterion that is superior to traditional information theory measures, this article develops a criterion function based on the minimum arithmetic and geometric divergence (MAGD) for image threshold segmentation.

In artificial settings like engineering buildings, the imaging conditions for cracks are very complex. It is often challenging to accurately distinguish between the background and the crack target in images using a simple threshold. Therefore, in this article, the proposed thresholding method is extended to multilevel to achieve the goal of distinguishing between the background and target in the crack image. For the issue of rapidly increasing algorithm time complexity caused by multilevel thresholding, an improved particle swarm optimization (PSO) algorithm is utilized in the implementation to acquire the optimal thresholds. In this way, the program execution time is reduced, satisfying the requirements of practical applications. The major contributions of this paper are as follows:

- A multilevel thresholding criterion for image segmentation based on the MAGD is presented.
- An improved particle swarm optimization algorithm combined with local stochastic perturbation (LSPIPSO) is presented.
- The LSPIPSO+MAGD multilevel thresholding method for crack image segmentation is proposed.
- The performance of the proposed method is compared with seven well-known multilevel thresholding methods using metrics such as RMSE, PSNR, SSIM, FSIM, and computational time.

The remainder of this article is structured as follows. The “Related work” section reviews the concepts of arithmetic-geometric divergence and the particle optimization algorithm. The image thresholding criteria and the multilevel thresholding method based on LSPIPSO are described in the “Proposed methodology” section. The “Experimental environment and evaluation metrics” section describes the experimental environment and performance evaluation metrics. The performance comparison analysis and discussion of results are described in the “Experimental results and discussion” section. The “Conclusions and future works” section summarizes this paper and outlines future research directions.

## Related work

In this section, we will review the concepts of arithmetic and geometric divergence measures, as well as the particle swarm optimization algorithm.

### Arithmetic-Geometric divergence

Let $\Xi =\{\Theta =(\theta _1,\theta _2,\dots ,\theta _n)|\theta _i\ge 0,\theta _1+\theta _2+\dots +\theta _n=1,i=1,2,\dots ,n;n\ge 2\}$ be a finite set of complete discrete probability distributions, $\textbf{A},\textbf{B}\in \Xi$. In the study of statistical distribution and information theory, Taneja proposed a new criterion for measuring the similarity (or dissimilarity) between different probability distributions^26^, which is also known as the divergence measure of information theory distance, i.e.

1 $$\begin{aligned} {D\left( \textbf{A}|\textbf{B}\right) =\sum _{i=1}^n\left[ \left( \frac{a_i+b_i}{2}\right) \log \left( \frac{a_i+b_i}{2a_i}\right) \right] } \end{aligned}$$

The divergence measure is referred to as Arithmetic-Geometric divergence, often abbreviated as AG divergence. The AG divergence compensates for the limitations of traditional divergences (such as the well-known Kullback-Leibler divergence, $\chi ^2$-Divergence, etc.) in quantifying distances in information theory. Based on Kullback-Leibler divergence, Li and Lee proposed the well-known minimum cross entropy image thresholding method^27^, which has been successfully applied in various fields. For complex scenarios, the effectiveness of this method was also confirmed through multilevel expansion^14,28^.

In many scenarios, the proportion of the image area occupied by the target pixels in crack images is highly uneven. This non-uniform distribution often makes it impossible to extract the crack target using simple thresholding methods. Here, we will develop a new image segmentation method utilizing AG divergence and evaluate its effectiveness in segmenting crack images.

### Particle swarm optimization

Particle swarm optimization (PSO) algorithm is an evolutionary computation technique^29,30^. Inspired by the regularity of bird swarming activities, Eberhart and Kennedy constructed a simplified model based on swarm intelligence in 1995 and proposed the PSO algorithm after observing bird swarming activities^29^. In the application of PSO algorithm to solve problems, information sharing among individuals in the group is utilized to facilitate an evolutionary process from disorder to order in the problem-solving space, aiming to achieve the optimal solution.

**Figure 1:**
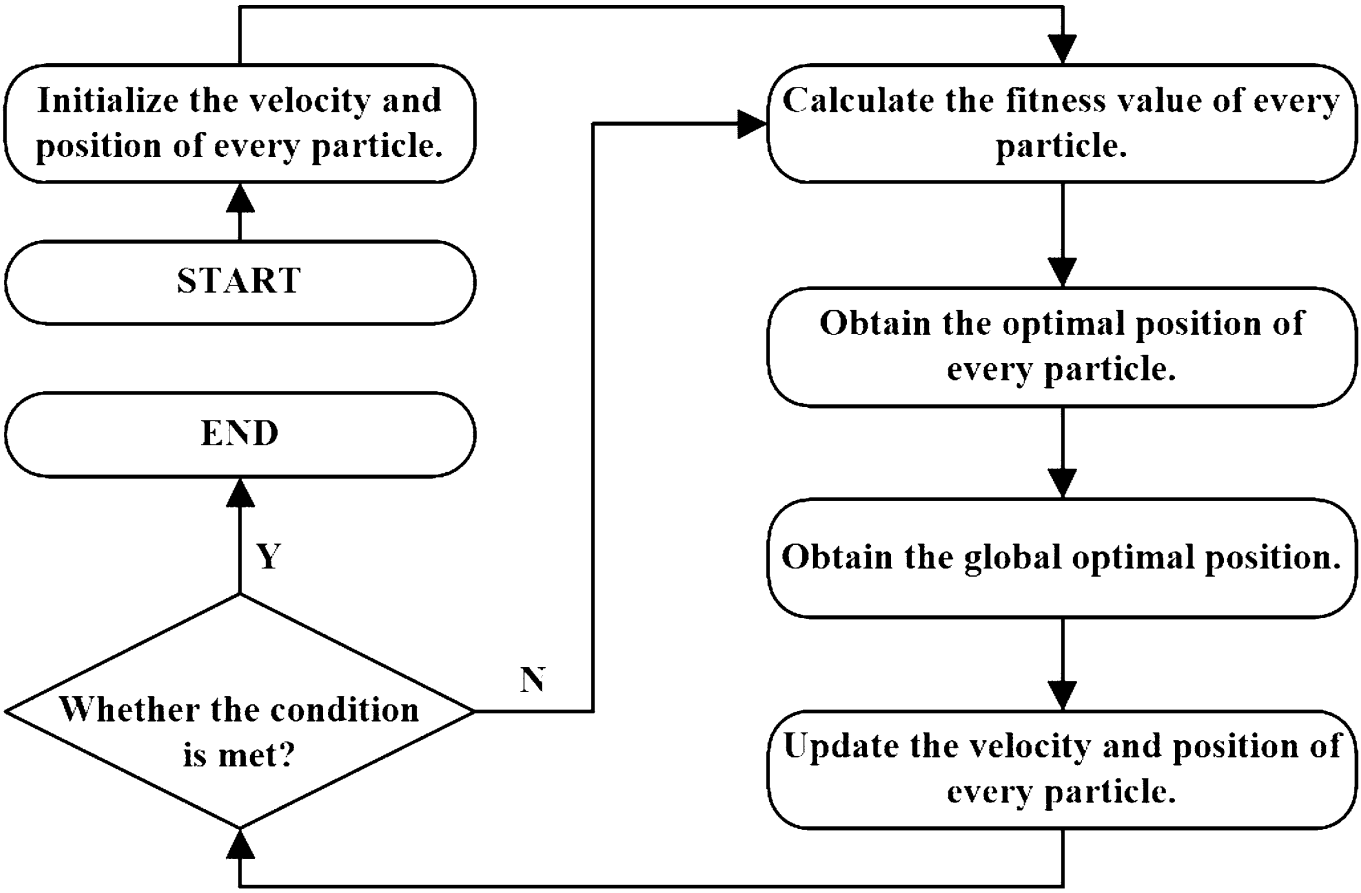
Flowchart of the basic PSO algorithm.

The basic flow of the PSO algorithm is illustrated in Figure 1. The most critical steps in the PSO algorithm involve updating the particle flight velocity and the particle position. For the classical particle swarm optimization algorithm, the following two equations describe how the particle velocity and position are updated.

2 $$\begin{aligned} v_{id}= & {} \kappa [v_{id}+c_1r_1(pBest_{id}-x_{id})+c_2r_2(gBest_d-x_{id})] \end{aligned}$$

3 $$\begin{aligned} x_{id}= & {} x_{id}+v_{id} \end{aligned}$$

Where $v_{id}$ is the velocity component of the *i*th particle in the *d*th dimension; $x_{id}$ is the position component of the *i*th particle in the *d*th dimension. $c_1$ and $c_2$ are learning factors, usually $c_1+c_2>4$. According to literature [], it is generally recommended to use $c_1=2.0$ and $c_2=2.1$. In our work, the values of $c_1$ and $c_2$ are also set in this way. $r_1$ and $r_2$ are two random decimals in the interval [0, 1]; $pBest_{id}$ represents the *d*th dimensional component of the optimal position of the *i*th individual; $gBest_d$ represents the *d*th dimensional component of the optimal position of the population. $\kappa$ is called the scaling factor, and the following definition is suggested^30^.

4 $$\begin{aligned} \kappa =\frac{1}{|2-\varphi -\sqrt{\varphi ^2-4\varphi }|} \end{aligned}$$

Here, $\varphi =c_1+c_2$, and if we take $c_1=2.0$ and $c_2=2.1$, then $\varphi = 0.729$.

In general, the steps of the PSO algorithm can be described as follows.

(1) Initialize all particles, i.e. assign initial values to the velocity and position of each particle, set the historical individual optimal position *pBest* of an individual to the current value, and set the optimal individual position in the group as the current group optimal position *gBest*.

(2) Calculate the value of the fitness function for each particle.

(3) If the current value of the fitness function is better than the historical optimal value, the *pBest* value is updated.

(4) If the current value of the fitness function is better than the historical optimal value of the population, the *gBest* value is updated.

(5) Update the velocity and position of the particles according to Eqs. (2)-(3).

(6) Determine if the end condition is satisfied. If the condition is not met, go to step (2) to continue iterating the algorithm.

## Proposed methodology

It is here that the details of the proposed method will be described in more detail in this section.

### Image thresholding criteria

Let the image to be segmented be $\textbf{I}=[\textbf{Ir},\textbf{Ig},\textbf{Ib}]_{m\times n \times c}$, where $m\times n$ denotes the size of the image, *c* is the number of channels (i.e., $c=3$ in the case of RGB images), and $\textbf{Ir}$, $\textbf{Ig}$, and $\textbf{Ib}$ denote the red, green, and blue-channel component images of the image $\textbf{I}$. When performing image thresholding segmentation, the three channel components of the image are segmented separately, and then the three components are synthesized after segmentation. Here, the red-channel component image $\textbf{Ir}$ of the image is used as an example to illustrate the thresholding criterion proposed in this paper.

Let the grayscale range of $\textbf{Ir}$ be $[0,1,2,\cdots ,L]$, and the estimated probability of each grayscale appearing in the image $\textbf{Ir}$ is $h_i (i=0,1,2,\cdots ,L)$, where *L* represents the maximum grayscale, $h_i=g_i/(m\times n)$, $g_i$ represents the number of pixels in an image with a grayscale level of *i*. In addition, if the optimal threshold found in the two-stage threshold segmentation of the image is *t*, then t divides the gray level of the image into two parts (i.e., two categories), assuming that these two parts are set as $\mathbf {C_0}=\{0,1,\cdots , t\}$ and $\mathbf {C_1}=\{t+1, t+2,\cdots , L\}$, respectively. For $\mathbf {C_0}$ and $\mathbf {C_1}$, where the class probabilities and the class means are defined over them, respectively, we have

5 $$\begin{aligned}{} & {} {P_0=\sum _{i=0}^{t}{h_i},\quad \quad \quad \quad P_1=\sum _{i=t+1}^{L}{h_i}} \end{aligned}$$

6 $$\begin{aligned}{} & {} {m_0=\frac{1}{P_0}\sum _{i=0}^{t}{ih_i},\quad \quad m_1=\frac{1}{P_1}\sum _{i=t+1}^{L}{ih_i}} \end{aligned}$$

Based on the above assumptions, according to the definition of AG divergence, the AG divergence values of images before and after segmentation can be calculated as follows.

7 $$\begin{aligned}{} & {} {D_0=\sum _{i=0}^{t}{\left( h_i\times \frac{i+m_0}{2}\right) }\log \left( \frac{i+m_0}{2i}\right) +\sum _{i=0}^{t}{\left( h_i\times \frac{i+m_0}{2}\right) \log \left( \frac{i+m_0}{2m_0}\right) }} \end{aligned}$$

8 $$\begin{aligned}{} & {} {D_1=\sum _{i=t+1}^{L}{\left( h_i\times \frac{i+m_1}{2}\right) }\log \left( \frac{i+m_1}{2i}\right) +\sum _{i=t+1}^{L}{\left( h_i\times \frac{i+m_1}{2}\right) \log \left( \frac{i+m_1}{2m_1}\right) }} \end{aligned}$$

Since the AG divergence is asymmetric, i.e., $D(\textbf{A}|\textbf{B})\ne D(\textbf{B}|\textbf{A})$, to consider the effectiveness of the AG divergence in image segmentation more comprehensively, we compute the divergence values before and after image segmentation in the form of $D(\textbf{A}|\textbf{B})+D(\textbf{B}|\textbf{A})$, resulting in Eqs. (7) and (8).

According to the basic principle of divergence in information theory, the smaller the divergence value between two systems, the more similar they are. In image segmentation, the more information the segmented image can retain from the original image, the better it can represent the original image. From this, the criterion function for image segmentation can be defined as follows.

9 $$\begin{aligned} {F(t^*)=\arg \min _{t\in [0,1,\cdots ,L]}{(D_0+D_1)}} \end{aligned}$$

Where $t^*$ denotes the optimal threshold, and $t^*$ can divide $[0,1,\cdots ,L]$ into two categories. In the original image, the pixel values less than or equal to $t^*$ are set to $x_0$, and the pixel values greater than $t^*$ are set to $x_1$. Here, $x_0$ and $x_1$ are two different integers between $[0,1,\cdots ,L]$. The two-level thresholding can be described as follows.

10 $$\begin{aligned} s(i,j)=\left\{ \begin{matrix} x_0&{} f(i,j)\le t^*\\ x_1&{} f(i,j)> t^* \end{matrix}\right. \end{aligned}$$

Here, *f*(*i*, *j*) represents the pixel value located at the coordinate (*i*, *j*) within the original image, while *s*(*i*, *j*) denotes the pixel value at the coordinate (*i*, *j*) within the segmented image.

In complex scenes, two-level thresholding sometimes cannot effectively extract the target. To solve this problem, multilevel extension is often used in image thresholding techniques to enhance the method’s performance. The generation conditions of crack images are very complex. During the experimental process, it was found that two-level thresholding sometimes fails to segment the crack target effectively. Therefore, the proposed thresholding method is extended here by multilevel thresholding to meet practical application requirements.

Suppose the thresholds obtained in multilevel thresholding of an image are $(t_1,t_2,\cdots ,t_k)$, where $0\le t_1<t_2<\cdots<t_k<L$. *k* thresholds can classify the image gray levels into $k+1$ classes. By imitating Eqs. (5)-(9), class probability $(P_0, P_1,\cdots ,P_k)$, class mean $(m_0, m_1,\cdots ,m_k)$, and divergence $(D_0, D_1,\cdots ,D_k)$ can be constructed. The function for calculating the total image AD value can be defined as follows.

11 $$\begin{aligned} {F(t_1,t_2,\cdots ,t_k)=D_0+D_1+\cdots +D_k} \end{aligned}$$

Eq. (11) is also used as the fitness function for swarm intelligence optimization algorithms in multilevel thresholding of images in the study of this paper. And so on, the criterion function of multilevel threshold segmentation can be obtained.

12 $$\begin{aligned} {F(t_1^*,t_2^*,\cdots ,t_k^*)=\arg \min _{t_1,t_2,\cdots ,t_k\in [0,1,\cdots ,L]}{F(t_1,t_2,\cdots ,t_k)}} \end{aligned}$$

After obtaining *k* optimal thresholds, according to the two-level thresholding classification method, a segmented image can be obtained by selecting $k+1$ different integers $(x_0,x_1,\cdots ,x_k)$ within the interval $[0,1,\cdots , L]$ based on the original image. The multilevel thresholding can be described as follows.

13 $$\begin{aligned} s(i,j)=\left\{ \begin{matrix} x_0 &{} f(i,j)\le t_1^*\\ x_1 &{} t_1^*< f(i,j)\le t_2^*\\ \cdots &{} \cdots \\ x_{k-1} &{} t_{k-1}^* < f(i,j) \le t_k^* \\ x_k &{} f(i,j)>t_k^* \\ \end{matrix}\right. \end{aligned}$$

Let $FB=floor(Max\_I/k)$, $Max\_I$ represents the maximum pixel value in the image, and for an 8-bit digital image, $Max\_I=255$. The function *floor*(*A*) represents a downward rounding function (the integer that is closest to *A*). Assuming that *k* thresholds are used to implement multilevel thresholding segmentation, the values of $(x_0,x_1,\cdots ,x_k)$ are set as follows in the algorithm implementation.

14 $$\begin{aligned} (x_0,x_1,\cdots ,x_{k-1},x_k)=(0,FB\times 1, FB\times 2, \cdots , FB\times (k-1), Max\_I) \end{aligned}$$

### Local stochastic perturbation

In multilevel image thresholding, the computation time increases exponentially as the number of thresholds increases. To effectively reduce the computation time, many methods apply evolutionary computation algorithms to solve this problem in image multilevel thresholding^15–20,31,32^. Since PSO has excellent performance in solving combinatorial optimization problems, it has been successfully applied to multilevel image thresholding^15,33^. PSO is a self-parallel evolutionary algorithm that is powerful for solving many optimization problems. However, like most evolutionary algorithms, it has its own shortcomings. For example, if there are multiple local extrema in solving a problem, the solution obtained by applying the PSO algorithm may be a local optimal solution rather than a global optimal solution. In our work, to avoid this phenomenon as much as possible, a local stochastic perturbation is added to each particle at the end of the execution of the conventional PSO algorithm to increase the diversity of individuals in the population, thus avoiding premature convergence or falling into local extreme traps of the algorithm. For each particle in the population, the local stochastic perturbation process is shown as Algorithm 1.

**Algorithm 1:**
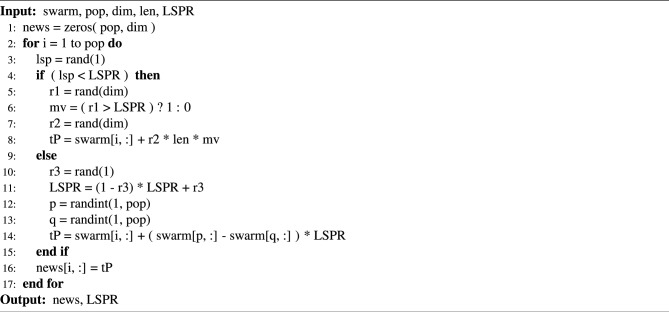
Local stochastic perturbation algorithm.

In Algorithm 1, ‘swarm’ represents the population space of candidate solutions to the problem; ‘pop’ and ‘dim’ denote the number of candidate solutions in the population space and the dimension of the solution, respectively; ‘len’ represents the size of the value interval for the candidate solution. ‘LSPR’ represents the Local Stochastic Perturbation Probability, which is a predetermined random decimal before the algorithm runs. ‘newS’ denotes a new population space. ‘rand(n)’ denotes the n-dimensional uniform random function for the interval [0,1]. ‘randint(1, n)’ is a function for generating uniform random integer from [1, *n*]. ‘p’ and ‘q’ are two integers chosen at random, and p $\ne$ q.

### Image segmentation algorithm

Using the image thresholding criteria and the extended PSO algorithm designed above for the segmentation of crack images, the flow of the algorithm is designed as follows. First, multilevel thresholding segmentation is implemented for the red, green and blue channel image of the original image, and the segmentation results of each channel image are obtained. Then, based on the segmentation results, the final segmentation results are obtained. The description of Algorithm 2 shows the flow of the multilevel thresholding image segmentation algorithm.

In Algorithm 2, ‘img’ represents the image to be segmented; ‘maxit’ denotes the maximum number of iterations allowed by the algorithm, ‘it’ represents the current iteration number of the algorithm; for ‘pop’, ‘dim’, ‘c1’, ‘c2’, ‘pBest’, and ‘gBest’, their meanings are the same as before.

**Algorithm 2:**
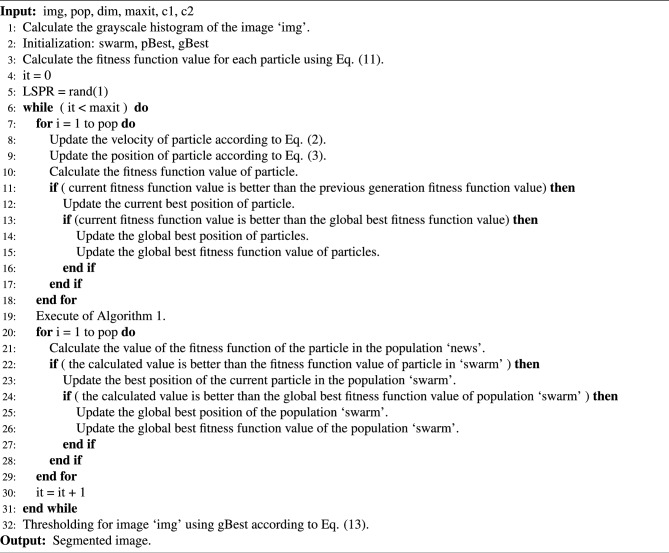
Multilevel thresholding algorithm for image segmentation.

In crack image segmentation, Algorithm 2 is applied to implement multilevel thresholding on the red, green, and blue channel images of the original image $\textbf{I}$, resulting in three segmented images: $\textbf{Sr}$, $\textbf{Sg}$, and $\textbf{Sb}$. The final multilevel thresholding segmented image $\textbf{S}$ can be obtained by synthesizing these three images.

15 $$\begin{aligned} {\textbf{S}=[\textbf{Sr},\textbf{Sg},\textbf{Sb}]} \end{aligned}$$

After multilevel thresholding, a significant amount of background information in the image is eliminated, which can enhance the visibility of the image target more effectively. This is very useful for extracting image targets. The extraction of image targets can be achieved through image binarization. In crack image segmentation, after multilevel thresholding, the crack is extracted by applying a binarization method. Assuming that the image obtained after binarization is $\textbf{B}$, the process of generating $\textbf{B}$ is as follows.

16 $$\begin{aligned} b(i,j)=\left\{ \begin{matrix} 0 &{} [s_r(i,j)<T] and [s_g(i,j)<T] and [s_b(i,j)<T] \\ 255 &{} otherwise \end{matrix}\right. \end{aligned}$$

Here, *b*(*i*, *j*) represents the pixel value at coordinates (*i*, *j*) in image $\textbf{B}$, $s_r(i,j)$ represents the pixel value at coordinates (*i*, *j*) in image $\textbf{Sr}$, $s_g(i,j)$ represents the pixel value at coordinates (*i*, *j*) in image $\textbf{Sg}$, $s_b(i,j)$ represents the pixel value at coordinates (*i*, *j*) in image $\textbf{Sb}$. *T* is a threshold used for binarization. In this study, *T* is determined as follows.

17 $$\begin{aligned} T=FB+1 \end{aligned}$$

**Figure 2:**
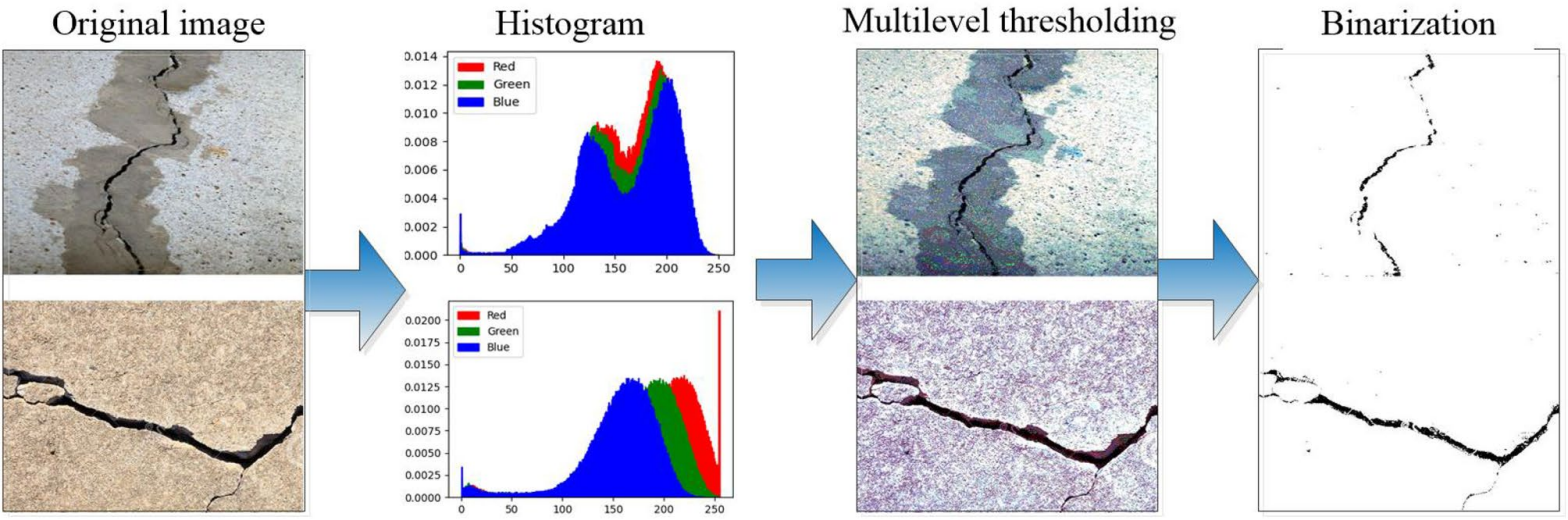
The process of crack image segmentation.

Here, the definition of *FB* aligns with the definition of *FB* in Eq. (14). Figure 2 summarizes the entire process of crack image segmentation.

## Experimental environment and evaluation metrics

To verify the effectiveness of the algorithm, we conducted experiments on the DeepCrack dataset^34^. DeepCrack is a public benchmark dataset with cracks in multiple scales and scenes to evaluate the crack detection systems. In our work, we conducted multiple tests on this dataset to compare the performance of the proposed algorithm with other cutting-edge methods for image multilevel thresholding, including particle swarm optimization (PSO)^15^, butterfly optimization (BFO)^16^, gases Brownian motion optimization (GBMO)^16^, exchange market algorithm (EMA)^17^, modified whale optimization algorithm (MWOA)^18^, hybrid whale optimization algorithm (HWOA)^19^, and cuckoo search optimization (CSO)^14^.

In our experiments, all algorithms are implemented using Python 3.9.13 with Pytorch 2.1.1 and run on a laptop with configurations of 32GB RAM, and Intel(R) Core(TM) i9-12900H CPU @2.50 GHz. The operating system is Windows 11 Home Edition 23H2. When running the algorithm, the maximum number of iterations allowed is set to 100 and the population size is set to 30. For the other parameters of the proposed method, they are set as described above. For the other parameters of the comparison method, the settings are the same as those described in the literature.

To evaluate the performance of the comparative algorithms, we used four evaluation metrics that are widely used in image quality analysis, namely RMSE, PSNR, SSIM^35^, and FSIM^36^. The definitions of these four metrics are as follows.

RMSE (Root Mean Square Error): RMSE is used to measure the degree of difference between the estimated values and the true values. The smaller the RMSE value, the smaller the difference between the two images.

18 $$\begin{aligned} {RMSE=\sqrt{\frac{1}{m\times n}\sum _{i=1}^m\sum _{j=1}^n\left[ s(i,j)-g(i,j)\right] ^2}} \end{aligned}$$

Here, $m\times n$ denotes the size of the image, *s*(*i*, *j*) and *g*(*i*, *j*) denote the pixel values at the coordinates (*i*, *j*) of the segmented image and the original image, respectively.

PSNR (Peak Signal to Noise Ratio): PSNR is used to measure the quality of an image. The higher the PSNR value, the greater the similarity between two images.

19 $$\begin{aligned} {PSNR=20\log _{10}{\left( \frac{MAX_\_I}{RMSE}\right) }} \end{aligned}$$

Where $MAX_\_I$ is the maximum pixel value in image *I*. For an 8-bit digital image, $MAX_\_I = 255$.

SSIM (Structural Similarity Index): The Structural Similarity Index is used to measure the structural similarity between two images. The closer the SSIM value is to 1, the more similar the two images are.

20 $$\begin{aligned} {SSIM=\frac{\left( 2 \mu _{x} \mu _{y}+C_{1}\right) \left( 2 \sigma _{x y}+C_{2}\right) }{\left( \mu _{x}^{2}+\mu _{y}^{2}+C_{1}\right) \left( \sigma _{x}^{2}+\sigma _{y}^{2}+C_{2}\right) }} \end{aligned}$$

Where, $\mu _x$ and $\mu _y$ denote the average pixel grayscale values of the segmented and original images; $\sigma _{x}$ and $\sigma _{y}$ are the standard deviations of the pixel grayscale values of the segmented image and the original image; $\sigma _{xy}$ is the covariance of pixel grayscale values between the segmented image and the original image, and $\sigma _{xy}=\frac{1}{N}\sum _{i=1}^N{(x_i-\mu _x)(y_i-\mu _y)}$; $C_1$ and $C_2$ are two constants. For an 8-bit digital image, $C_1=(0.01\times 255)^2$ and $C_2=(0.03\times 255)^2$ are often used.

FSIM (Feature Similarity Index): The Feature Similarity Index is used to measure the feature similarity between two images. The higher the FSIM value, the higher the feature similarity between two images.

21 $$\begin{aligned} {FSIM=\frac{\sum _{x\in \Omega }[S_L(x)\cdot PC_m(x)]}{\sum _{x\in \Omega }PC_m(x)}} \end{aligned}$$

Where, $S_L(x)=S_{PC}(x)\cdot S_G(x)$, $S_{PC}(x)=\frac{2PC_1(x)\cdot PC_2(X)+T_1}{PC_1^2(x)+PC_2^2(x)+T_1}$, $S_G(x)=\frac{2G_1(x)\cdot G_2(x)+T_2}{G_1^2(x)+G_2^2(x)+T_2}$, $G_x=\sqrt{G_x(x)^2+G_x(y)^2}$, $PC(x)=\frac{\sum _j{E_{\theta _j}}(x)}{\varepsilon +\sum _n\sum _j A_{n,\theta _j}(x)}$, $PC_m(x)=max(PC_1(x), PC_2(x))$. *PC*(*x*) represents the phase congruency feature of image, *G*(*x*) represents the gradient features of image, $S_{PC}(x)$ represents the similarity of the phase congruence features, $S_G(x)$ represents the similarity of the gradient features, and $S_L(x)$ represents the fusion similarity between *PC*(*x*) and *G*(*x*). The detailed principle and calculation process of FSIM can be found in the original reference^36^ by Zhang et al.

**Figure 3:**
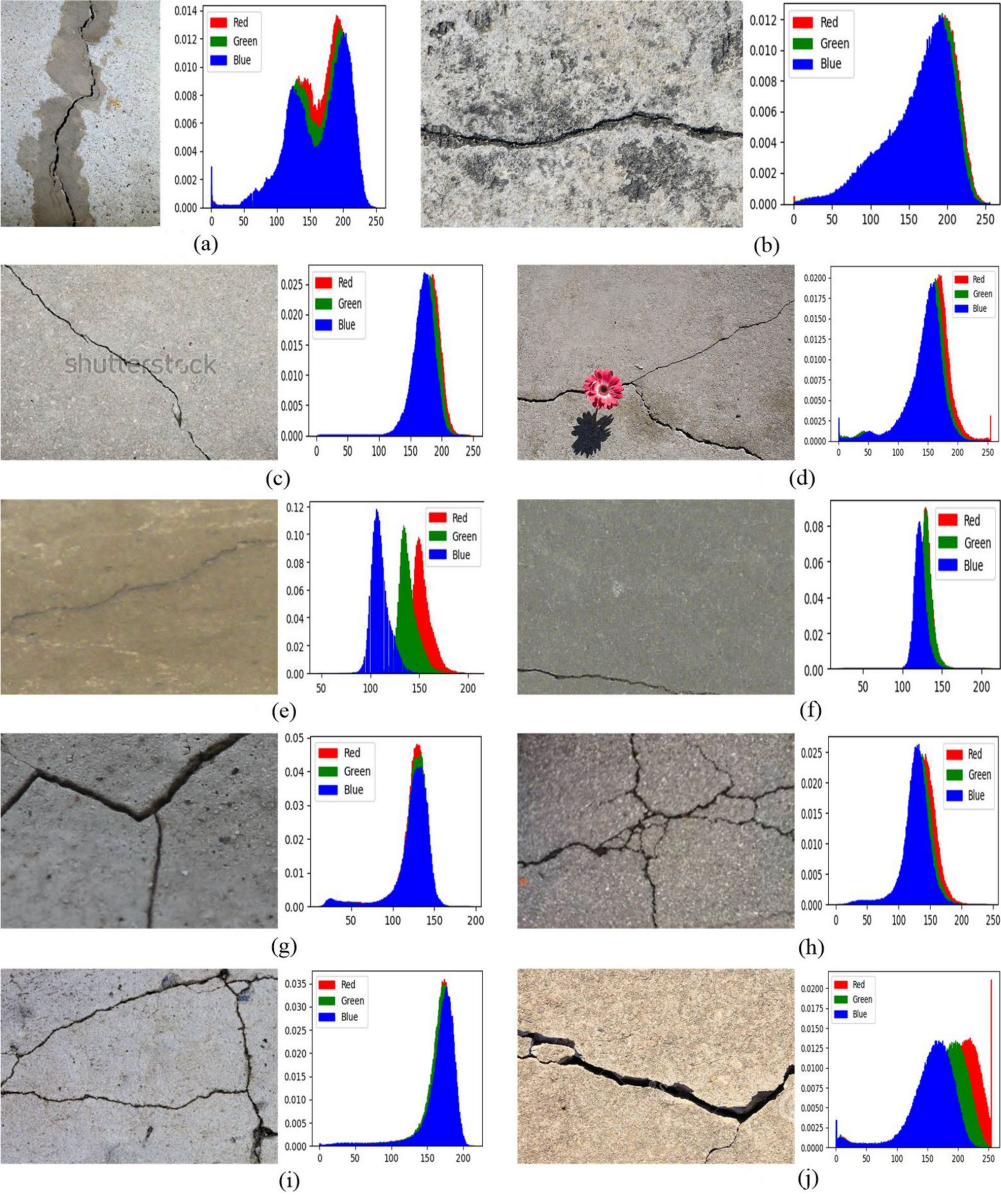
The test images and their histograms.

We use the images in the DeepCrack dataset to validate the effectiveness of the proposed method. The DeepCrack dataset contains 537 images. It is not possible to list the results of all images in the paper. Therefore, we randomly selected 10 highly representative images of various types to demonstrate the different performance of these methods. The 10 images and the grayscale histograms of their red, green, and blue channel images are shown in Figure 3. These images are named ‘11220-1.jpg’, ‘11266-3.jpg’, ‘11295.jpg’, ‘11308-2.jpg’, ‘IMG24-1.jpg’, ‘IMG109-1.jpg’, ‘7Q3A9060-9.jpg’, ‘11112.jpg’, ‘11134-2.jpg’, and ‘11190-2.jpg’ in DeepCrack dataset. For ease of reference, these images have been renamed as ‘img1’, ‘img2’, ‘img3’, ‘img4’, ‘img5’, ‘img6’, ‘img7’, ‘img8’, ‘img9’, and ‘img10’ in this paper. The size of ‘img1’ is $384\times 544\times 3$, the sizes of the other images are $544\times 384\times 3$.

For cracked images, there may be single or multiple cracks present against simple or complex backgrounds (e.g., contaminated by foreign objects, etc.). Figure 3 contains various types of crack images in DeepCrack dataset and common scenarios. For example, images with a single crack (such as ‘img1’, ‘mg2’, ‘img3’, ‘img5’, ‘img6’, ‘img10’) and images with multiple cracks (such as ‘img4’, ‘img7’, ‘img8’, ‘img9’). Images with simple backgrounds (such as ‘img6’, ‘img7’, ‘img8’, ‘im9’, ‘img10’), images with complex backgrounds (such as ‘img1’, ‘img2’, ‘img3’, ‘img4’), and images with contaminated backgrounds (such as ‘img5’). In addition, Figure 3 also shows that the crack images have complex backgrounds, and the cracks occupy a relatively small area of the entire image. In terms of the pixel grayscale histogram distribution of the images, the histograms of these images often show a single-peak distribution due to the significant proportion of background pixels. It is difficult to identify crack targets by analyzing histograms.

## Experimental results and discussion

In the description of the experimental results, the improved PSO method proposed in this paper is referred to as LSPIPSO. The proposed criterion for thresholding the image is called the MAGD criterion (Minimum Arithmetic-Geometric divergence criterion). In the experiments, LSPIPSO+MAGD, PSO+MAGD, BFO+MAGD, GBMO+MAGD, EMA+MAGD, MWOA+MAGD, HWOA+MAGD, and CSO+MAGD were utilized to conduct multilevel thresholding segmentation on the test images, and the performance of each method was compared.

### Segmentation results and analysis

**Figure 4:**
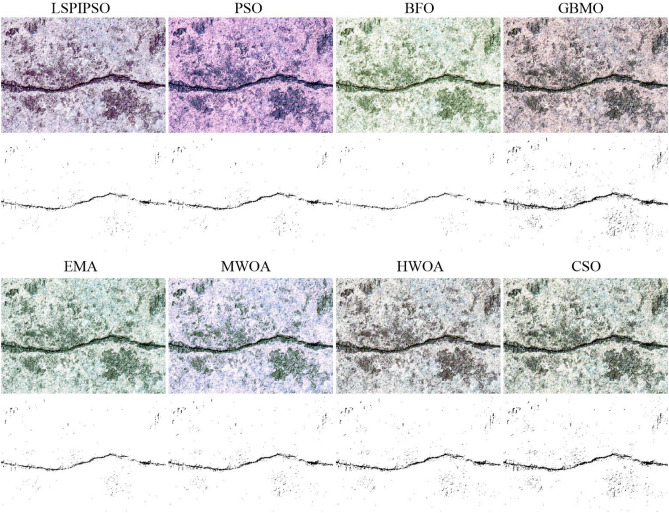
The segmented results of ‘img2’ by different algorithms with 9 thresholds.

**Figure 5:**
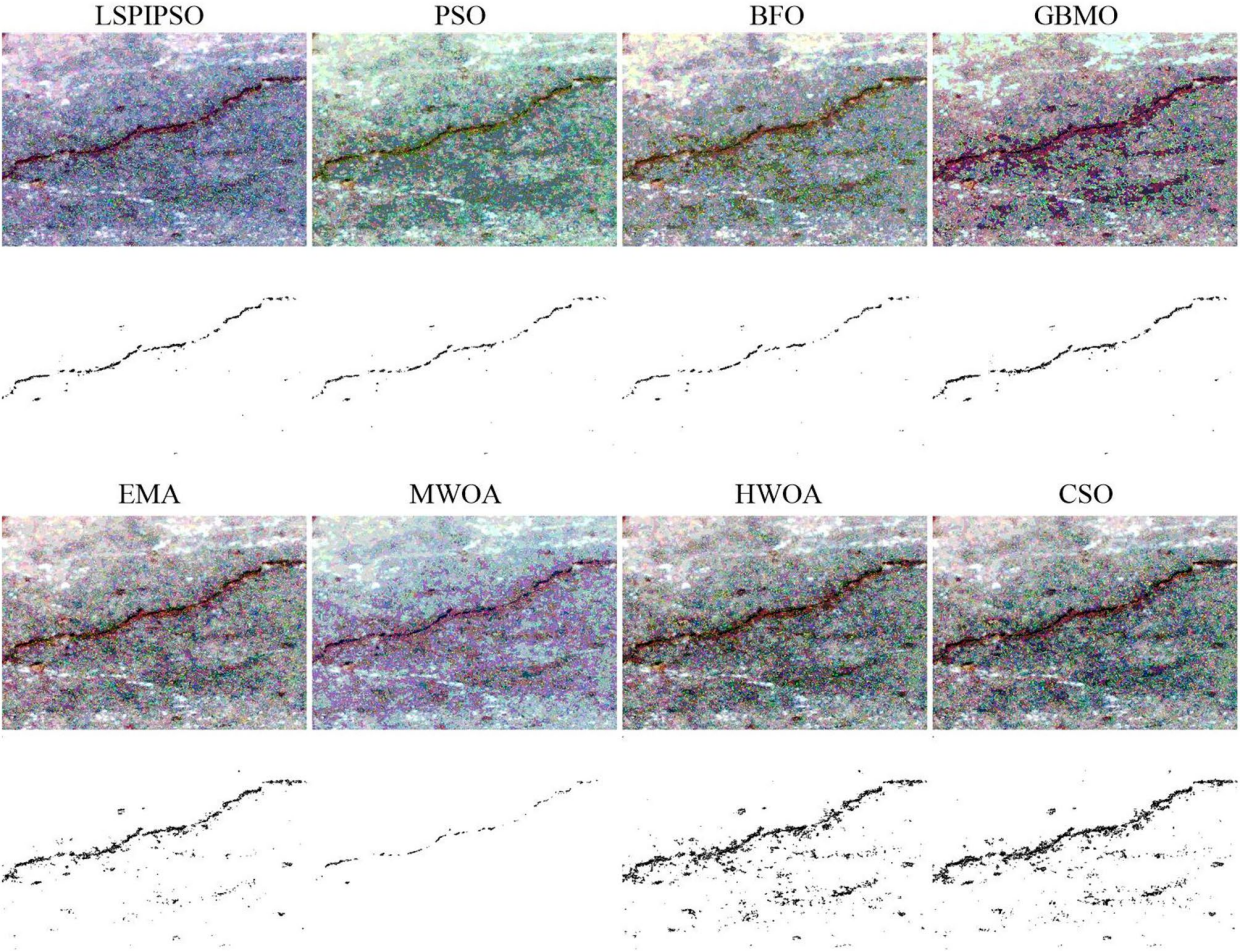
The segmented results of ‘img5’ by different algorithms with 7 thresholds.

**Figure 6:**
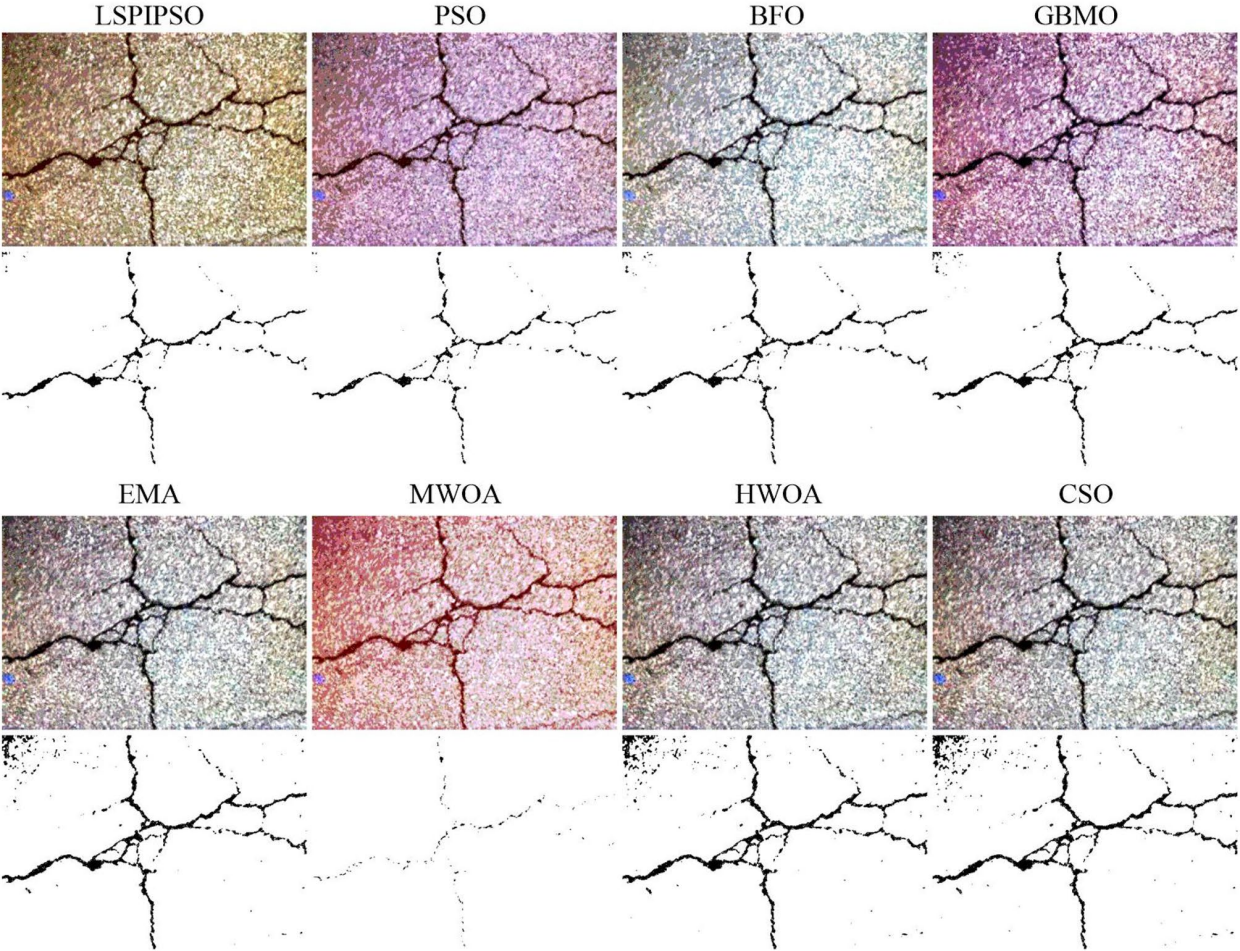
The segmented results of ‘img8’ by different algorithms with 5 thresholds.

In this subsection, we use ‘img2’, ‘img5’, and ‘img8’ as representatives to describe the multilevel thresholding results of crack images. The reason for selecting these images is that in Figure 3, it is evident that the background of ‘img2’ is highly complex, whereas the background of ‘img5’ is contaminated, making it challenging to separate the cracks in these two images from the background. The image ‘img8’ contains multiple complex cracks, making it challenging to segment them. Figures 4, 5, 6 display the segmentation results of these three test images. The image segmentation results with a slightly simpler background can be seen in Figure 2. The results in Figure 4 were obtained by different algorithms with 9 thresholds. Figure 2 contains the results of the algorithm presented in this paper for segmenting ‘img1’ and ‘img10’ using 9 and 5 thresholds, respectively.

From Figures 4, 5, 6, we can see that multilevel thresholding significantly reduces the background information of the original image and better highlights the crack target to be focused on. For crack images, selecting an appropriate number of thresholds can eliminate most of the background information in the image and segment the crack target. It is also found that for images with more complex cracks (such as ‘img2’ and ‘img5’), more thresholds are selected to segment the target through multilevel thresholding and binarization. For images with simple backgrounds (such as ‘img8’ and ‘img10’), a few thresholds can be selected to segment the target. From Figures 4, 5, 6, it can also be seen that the EMA, HWOA, and CSO algorithms exhibit under-segmentation in the binarization results, while the MWOA algorithm shows an over-segmentation phenomenon. The other methods achieved similar segmentation results by eliminating most of the background information and segmenting the target using multilevel thresholding and binarization.

### Objective evaluation and analysis

In this subsection, to evaluate the performance of each algorithm more objectively, we selected 2, 3, 5, 7, 9, 11, 15, and 20 thresholds to perform multilevel thresholding on the test images. Subsequently, the algorithms are assessed using the objective evaluation metrics, namely, the RMSE, PSNR, SSIM, and FSIM, mentioned earlier. Here, we calculate the averages obtained by each algorithm with 20 independent runs for each number of thresholds on these evaluation metrics and their mean rankings. These statistical results are recorded in 10 Tables. Due to the large amount of data, for the sake of clarity, we have included these data as supplementary materials in the “Additional information” section. For specific details about these data, please refer to the “Supplementary Information” in the “Additional information” section.

**Figure 7:**
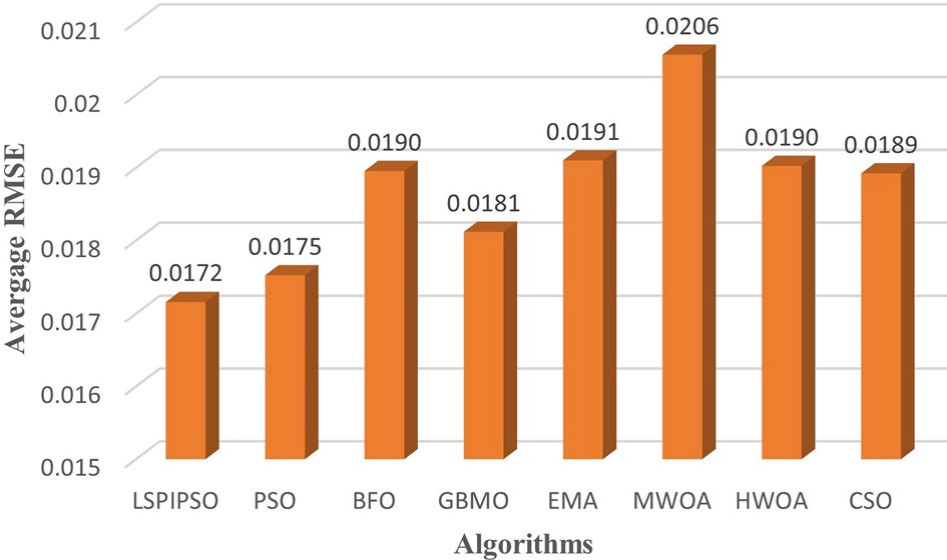
The average RMSE values obtained by different algorithms on all test images.

**Figure 8:**
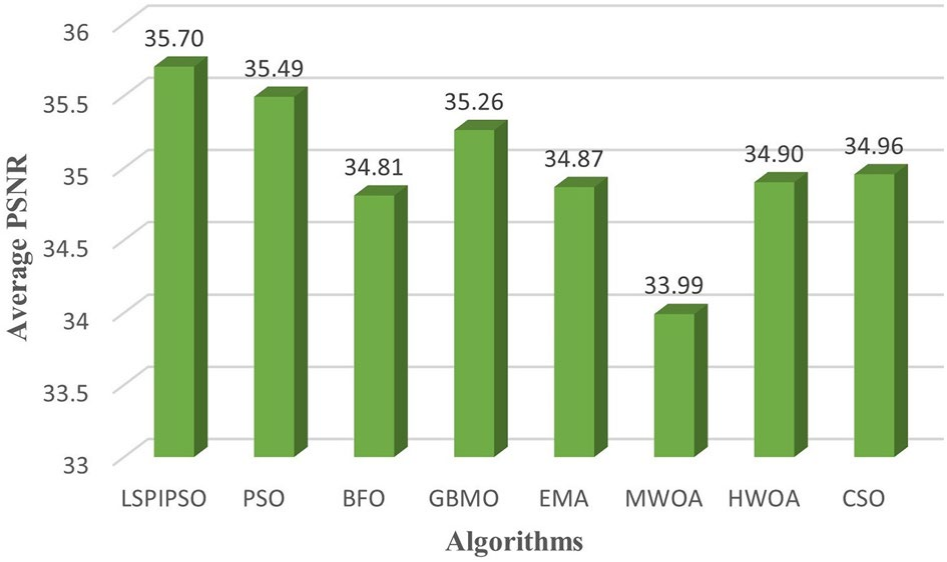
The average PSNR values obtained by different algorithms on all test images.

**Figure 9:**
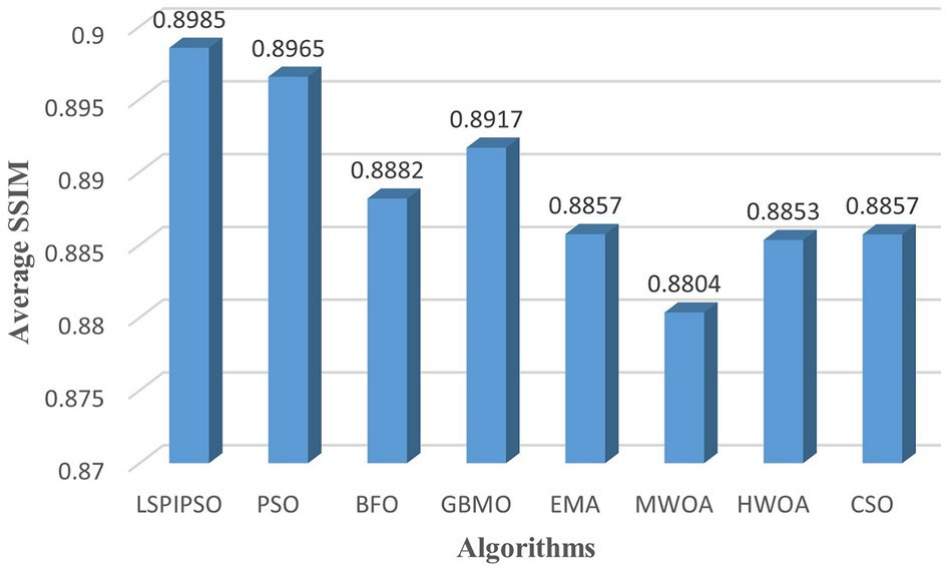
The average SSIM values obtained by different algorithms on all test images.

**Figure 10:**
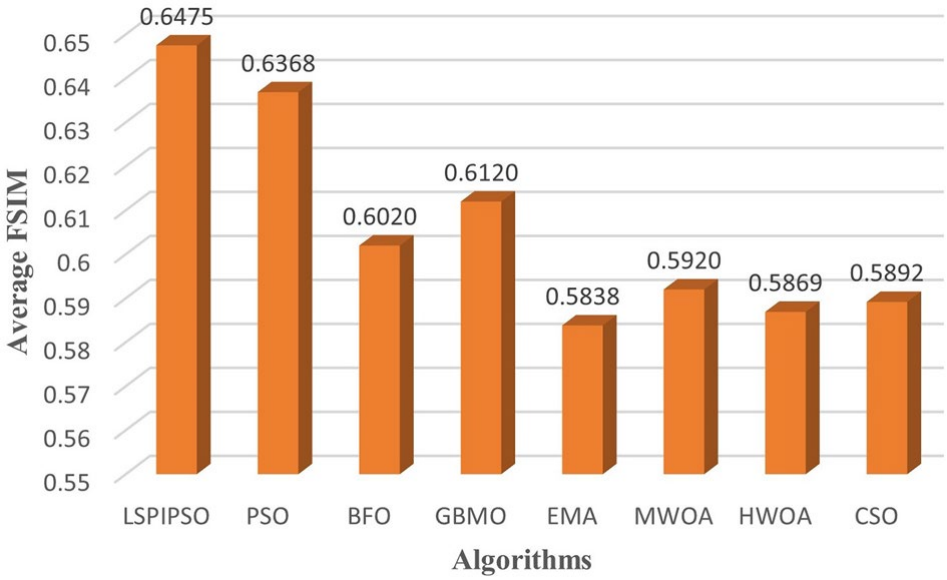
The average FSIM values obtained by different algorithms on all test images.

Based on these data, the average values of each metric obtained by different algorithms on all test images subjected to multilevel thresholding are shown in Figures 7, 8, 9, 10. This display is more intuitive. As can be seen from Figures 7, 8, 9, 10, the proposed algorithm achieves the smallest average on RMSE and the largest average on PSNR, SSIM, and FSIM.

**Figure 11:**
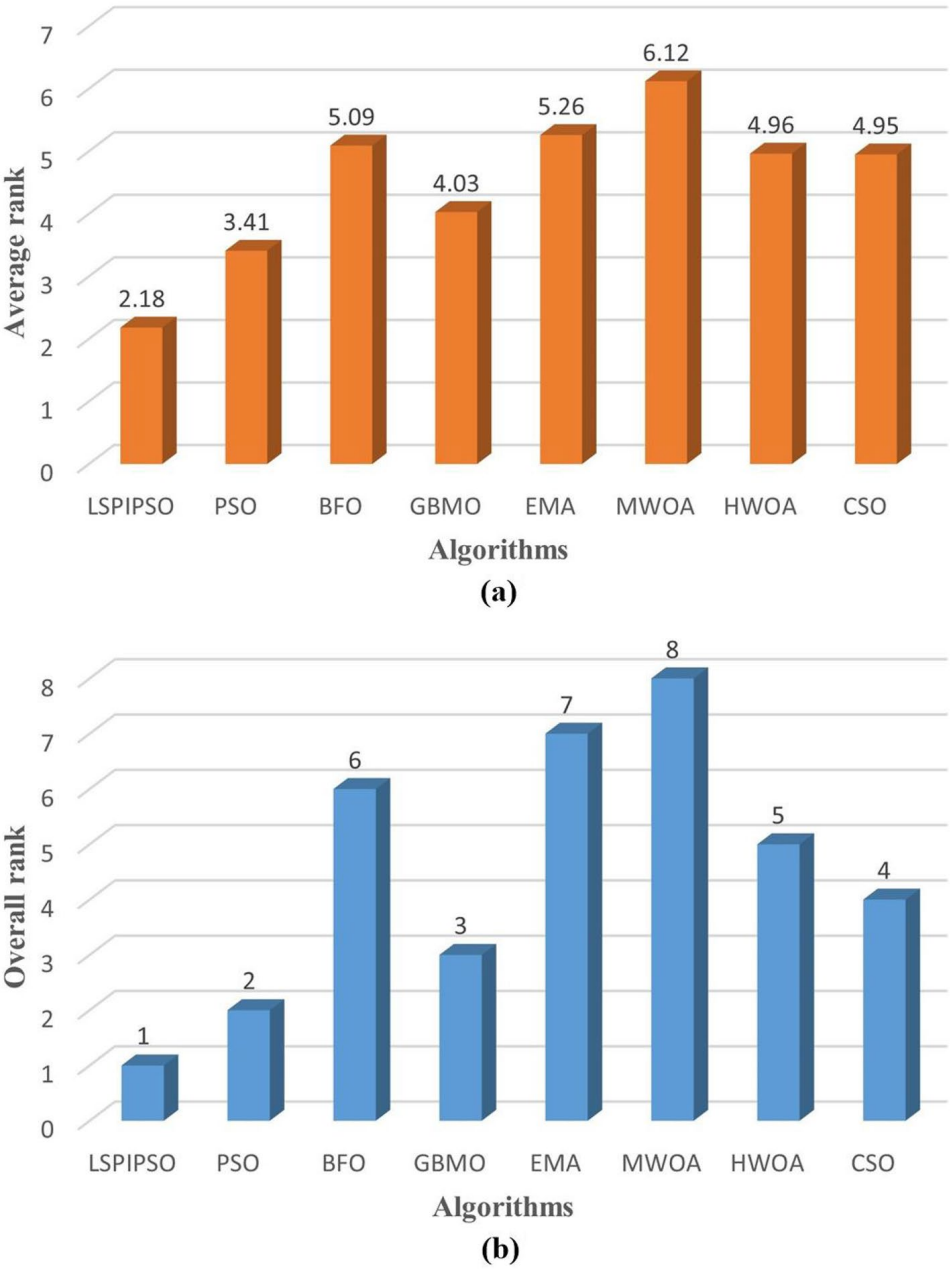
The average and overall ranking results of each algorithm for all metrics on all test images. (**a**) The average ranking. (**b**) The overall ranking.

The comprehensive ranking of each algorithm based on the evaluation metrics provides a thorough representation of the algorithm’s performance. Figure 11 displays the comprehensive ranking of each algorithm based on evaluation metrics. The average ranking and overall ranking are displayed in Figure 11. As can be seen in Figure 11, the ranking order from low to high is LSIPSO, PSO, GBMO, CSO, HWOA, BFO, EMA, and MWOA.

**Figure 12:**
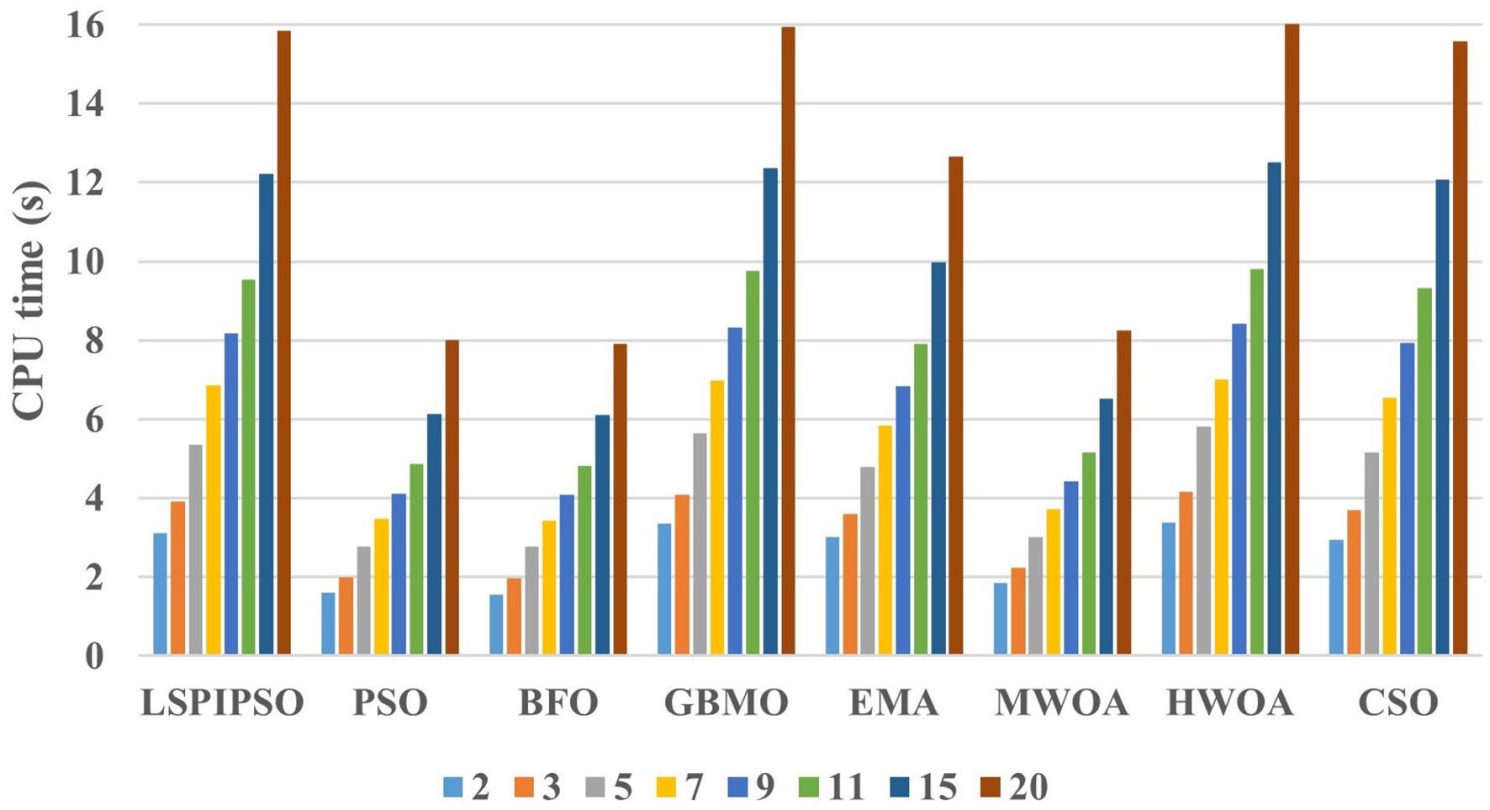
The average CPU time (s) of each algorithm at different number of thresholds on all test images.

To assess the practical usability of an algorithm, time performance is also a crucial indicator. For the 8 algorithms, i.e., LSPIPSO, PSO, BFO, GBMO, EMA, MWOA, WHOA, and CSO, the time complexities are $O(MAXIT\times POP + MAXIT\times POP)$, $O(MAXIT\times POP)$, $O(MAXIT\times POP)$, $O(MAXIT\times POP + MAXIT\times POP)$, $O(MAXIT\times POP + MAXIT\times POP + MAXIT\times POP)$, $O(MAXIT\times POP)$, $O(MAXIT\times POP + MAXIT\times POP)$, and $O(MAXIT\times POP + MAXIT\times POP)$, respectively. Here, *MAXIT* represents the maximum number of iterations allowed by the algorithm, and *POP* represents the population size (i.e., the number of candidate solutions to the problem). When the constant coefficients are removed, the time complexity of each algorithm is actually $O(MAXIT\times POP)$. Figure 12 shows the average CPU time consumed by each algorithm for multilevel thresholding of the test images at different numbers of thresholds.

As depicted in Figure 12, the CPU time of each algorithm increases significantly as the number of thresholds increases. From Figure 12, we can observe that the CPU time of LSPIPSO, GBMO, EMA, HWOA, and CSO algorithms is higher than that of other algorithms in multilevel threshold segmentation on the test images. The CPU time of the proposed method LSPIPSO is higher than that of PSO, BFO, and MWOA algorithms, but in most cases, it is lower than that of GBMO and HWOA algorithms. The CPU time of the EMA and CSO algorithms is comparable to that of the proposed method. From this perspective, the proposed method remains competitive in terms of time performance.

### Algorithm convergence analysis

**Figure 13:**
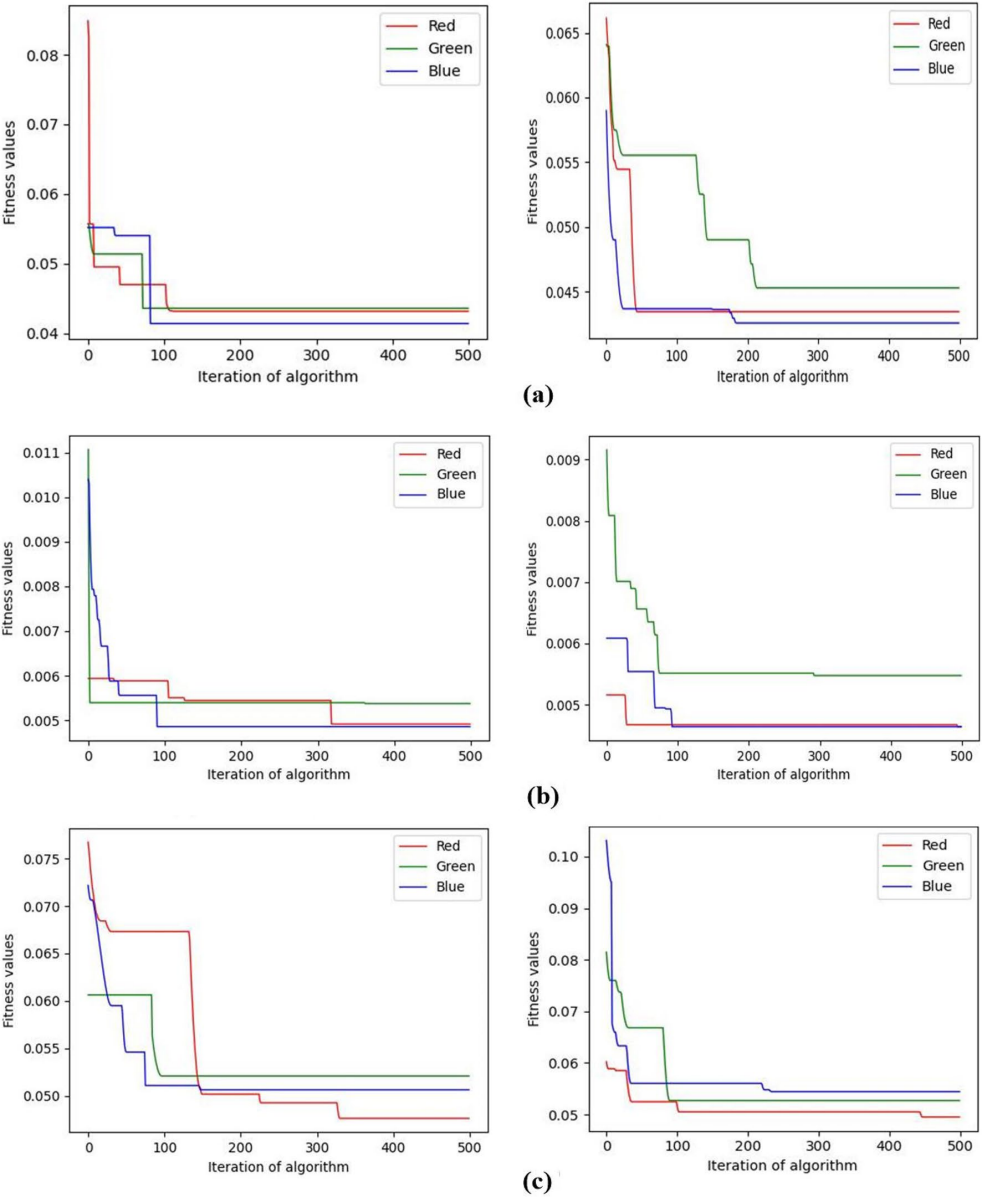
The convergence curve of fitness function of the proposed algorithm under different population size. The population size of the left subgraph is 30, and the right one is 60. (**a**) The convergence curve of fitness function of img2 with 9 thersholds. (**b**) The convergence curve of fitness function of img5 with 7 thersholds. (**c**) The convergence curve of fitness function of img8 with 5 thersholds.

Convergence analysis of algorithms is an important aspect of evaluating algorithm performance. For swarm intelligence optimization algorithms, this performance can be analyzed from aspects such as algorithm convergence speed, stability, etc. The convergence speed and stability of swarm intelligence optimization algorithms can be influenced by factors such as algorithm iteration times and population size. To examine the convergence performance of the algorithm proposed in this paper, experiments were conducted with the number of iterations of the algorithm set to 500 and the population size set to 30 and 60, respectively. Figure 13 illustrates the experimental results. Figure 13 shows the algorithm convergence curves for the test images ‘img2’ with 9 thresholds, ‘img5’ with 7 thresholds, and ‘img8’ with 5 thresholds. For each test image in this experiment, the convergence curves of their red, green, and blue channel images are all shown in Figure 13. As can be seen from Figure 13, the fitness function value decreases in a stepwise fashion, with a more significant decrease in the first 100 generations, irrespective of whether the population size is 30 or 60. Through experiments, it was found that as the population size increases, the algorithm’s convergence speed becomes faster in the vast majority of cases. In addition, a shortcoming of the algorithm proposed in this paper is also evident from this experiment. In some cases, the algorithm does not converge to the extremes quickly, as illustrated in Figure 13c. This area also requires attention for enhancing algorithm performance in future research.

## Conclusions and future works

Crack detection is of great importance in modern architecture and life. It can not only ensure the safety and stability of buildings, but also improve the life and performance of buildings. With the development of digitalization and intelligent technology, crack detection based on image thresholding segmentation technology has become an important means of intelligent management in buildings. By using image thresholding segmentation technology, we can separate the crack region from the background region in the image to more accurately measure and evaluate the condition and position of the crack. This can provide an important reference for the maintenance and reinforcement of buildings, and ensure the accuracy and effectiveness of maintenance work.

Arithmetic-geometric divergence is a measurement method that can be used to measure the uneven distribution of pixel values in images. It combines the advantages of arithmetic mean and geometric mean and can better capture the local characteristics of the image. Based on this divergence criterion, we propose a multilevel image thresholding method for crack detection. To reduce the computation time, the proposed method combines particle swarm optimization (PSO) algorithm to obtain optimal thresholds. In the meantime, the method combines a local stochastic perturbation to overcome the shortcomings of the PSO. In order to investigate the performance of the proposed method (LSPIPSO), a series of experiments have been performed on crack image dataset. Seven well-known competing methods for multilevel thresholding image segmentation, including Particle Swarm Optimization (PSO), Butterfly Optimization (BFO), Gas Brownian Motion Optimization (GBMO), Exchange Market Algorithm (EMA), Modified Whale Optimization Algorithm (MWOA), Hybrid Whale Optimization Algorithm (HWOA), and Cuckoo Search Optimization (CSO) are selected for parallel comparison. A comprehensive evaluation was performed using subjective methods such as visual observation, as well as objective methods such as RMSE, PSNR, SSIM, FSIM and computation time. The experimental results show that the proposed method outperforms several competing methods with respect to these metrics in many cases.

For further studies, the proposed method can be applied to various real-world applications, such as agricultural, medical, and industrial image segmentation tasks. The proposed improved PSO algorithm can also be used to solve other optimization problems. The local stochastic perturbation can also be integrated into other population optimization algorithms to improve their performance.

### Supplementary Information

Supplementary Information.

## Data Availability

The datasets generated and/or analyzed during the current study are available in the DeepCrack repository, [https://github.com/yhlleo/DeepCrack/].
