# Supplementary material for: Multilevel thresholding with divergence measure and improved particle swarm optimization algorithm for crack image segmentation
Source: Sci Rep. 2024 Apr 1;14:7642. doi: 10.1038/s41598-024-58456-2 (PMC10984966; doi:10.1038/s41598-024-58456-2)
Supplement: Supplementary file 1 — Supplementary Information. [file 41598_2024_58456_MOESM1_ESM.pdf]

Supplemental Material

The supplemental material includes 10 Tables, namely, Table S1-S10.

In Tables S1-S10, 'numTh' represents the number of thresholds. When 'numTh' is set to 2, 3, 5, 7, 9, 11, 15, and 20, multilevel thresholding experiments are performed on the test images. In a Table, the statistical mean values and the ranking of the evaluation metrics obtained by different algorithms on a test image are recorded. For statistical mean values of each metric, they are obtained by each algorithm over 20 runs independently. For each value of 'numTh', the top row records the statistical mean values of the metric, and the bottom row records the algorithm ranking on this metric. Meanwhile, the average ranking and overall ranking of each metric for different algorithms are also record in every Table.

From Tables S1-S10, we can observe that in most cases, the results obtained by the algorithm LSPIPSO proposed in this paper are superior to those of other compared algorithms. The data in Tables S1-S10 is the basic data for generating Figures 7-11 in the paper.

Metric	numTh	LSPIPSO	PSO	BFO	GBMO	EMA	MWOA	HWOA	CSO
RMSE	2	0.013579765	0.013627215	0.013611689	0.013609513	0.01363374	0.014366043	0.01363374	0.01363374
		1	4	3	2	5	8	6	7
	3	0.015382529	0.01549218	0.015473573	0.015364363	0.01551602	0.015747819	0.015519218	0.015517113
		2	4	3	1	5	8	7	6
	5	0.017902501	0.017847427	0.018212782	0.018395705	0.018542387	0.018538306	0.018552707	0.018555867
		2	1	3	4	6	5	7	8
	7	0.018251417	0.018556913	0.018938111	0.019327203	0.019840546	0.019780471	0.019807125	0.019837192
		1	2	3	4	8	5	6	7
	9	0.01795	0.018745	0.019952739	0.019877134	0.020725828	0.02076011	0.020672652	0.020691491
		1	2	4	3	7	8	5	6
	11	0.017254	0.018224	0.020496275	0.020337297	0.021388378	0.021354353	0.021339695	0.021337363
		1	2	4	3	8	7	6	5
	15	0.016523	0.016744	0.020116666	0.019425795	0.022293698	0.022197213	0.022225116	0.022171962
		1	2	4	3	8	6	7	5
	20	0.013688	0.014015	0.016819988	0.01605766	0.02115505	0.021140743	0.020987661	0.020917333
		1	2	4	3	8	7	6	5
	Average rank	1.25	2.375	3.5	2.875	6.875	6.75	6.25	6.125
	Overall rank	1	2	4	3	8	7	6	5
PSNR	2	37.33507967	37.3026339	37.31329311	37.31474973	37.29983082	36.8249565	37.29983082	37.29983082
		1	4	3	2	5	8	6	7
	3	36.25524373	36.19144248	36.20314813	36.26748123	36.18209352	36.05282949	36.18028062	36.18147899
		2	4	3	1	5	8	7	6
	5	34.9181232	34.95818981	34.79057246	34.70288581	34.63524069	34.63761273	34.63043757	34.62897388
		2	1	3	4	6	5	7	8
	7	34.73528169	34.59959988	34.44604164	34.27442745	34.04749559	34.0735713	34.06207672	34.04895715
		1	2	3	4	8	5	6	7
	9	34.8969946	34.53206136	33.99399364	34.02812928	33.66822989	33.65409504	33.69043226	33.68261203
		1	2	4	3	7	8	5	6
	11	35.23999906	34.76632626	33.74997742	33.82864231	33.39487298	33.40855823	33.41453421	33.41565386
		1	2	4	3	8	7	6	5

	**15**	35.61829505	35.47748834	33.91971858	34.22555335	33.03471521	33.07275086	33.06138538	33.0822236
		1	2	4	3	8	6	7	5
	**20**	37.27411967	37.05258093	35.4540459	35.87508654	33.49018748	33.49643883	33.55893496	33.58784059
		1	2	4	3	8	7	6	5
	**Average rank**	1.25	2.375	3.5	2.875	6.875	6.75	6.25	6.125
	**Overall rank**	1	2	4	3	8	7	6	5
**SSIM**	**2**	0.935186784	0.93488438	0.934460054	0.934804526	0.934437368	0.929530866	0.934437368	0.934437368
		1	2	4	3	5	8	6	7
	**3**	0.92887556	0.924152797	0.927705909	0.92868954	0.927071938	0.926960236	0.92703946	0.927060911
		1	8	3	2	4	7	6	5
	**5**	0.913990379	0.914745382	0.911867379	0.910822268	0.908779334	0.90979603	0.908693534	0.908670545
		2	1	3	4	6	5	7	8
	**7**	0.912304648	0.910291257	0.907531937	0.905083247	0.900407268	0.901219646	0.900669232	0.900441346
		1	2	3	4	8	5	6	7
	**9**	0.914705868	0.909588952	0.900691364	0.901529957	0.89460615	0.89465215	0.895011205	0.894898261
		1	2	4	3	8	7	5	6
	**11**	0.919265919	0.912996783	0.896883921	0.89836453	0.890211004	0.890621348	0.890560366	0.890651426
		1	2	4	3	8	6	7	5
	**15**	0.923516888	0.921447707	0.899063053	0.904269383	0.884087263	0.884860729	0.884599582	0.885050515
		1	2	4	3	8	6	7	5
	**20**	0.936056398	0.934266521	0.918257124	0.922950059	0.891730638	0.891808718	0.892974121	0.893398407
		1	2	4	3	8	7	6	5
	**Average rank**	1.125	2.625	3.625	3.125	6.875	6.375	6.25	6
	**Overall rank**	1	2	4	3	8	7	6	5
**FSIM**	**2**	0.330155977	0.33144628	0.324587452	0.328588609	0.325598493	0.339409614	0.325598493	0.325598493
		3	2	8	4	5	1	6	7
	**3**	0.40426959	0.405705277	0.396467053	0.400065869	0.387648236	0.405082526	0.387158254	0.387496742
		3	1	5	4	6	2	8	7
	**5**	0.442160285	0.447102373	0.43201561	0.421991865	0.396226236	0.415919531	0.395211072	0.394731013
		2	1	3	4	6	5	7	8
	**7**	0.482726485	0.478737254	0.466116132	0.448329619	0.399732131	0.417774679	0.402800265	0.400393644
		1	2	3	4	8	5	6	7
	**9**	0.540626656	0.523817052	0.458343254	0.47298537	0.401967985	0.41438098	0.407232467	0.406108661
		1	2	4	3	8	5	6	7
	**11**	0.589652117	0.55508922	0.449582866	0.475261242	0.401653968	0.418257574	0.406267433	0.408698241
		1	2	4	3	8	5	7	6
	**15**	0.637810062	0.626295665	0.483127353	0.53659285	0.397659815	0.417137589	0.405721639	0.414160677
		1	2	4	3	8	5	7	6
	**20**	0.66399082	0.654588518	0.593668585	0.612586795	0.406088399	0.426349044	0.420624428	0.43640908
		1	2	4	3	8	6	7	5
	**Average rank**	1.625	1.75	4.375	3.5	7.125	4.25	6.75	6.625
	**Overall rank**	1	2	5	3	8	4	7	6

Table S1. The comparison of statistical results of the values of performance evaluation metrics for all algorithms on test image 'img1'.

Metric	numTh	LSPIPSO	PSO	BFO	GBMO	EMA	MWOA	HWOA	CSO
**RMSE**	**2**	0.016789231	0.016779616	0.01671361	0.016702685	0.016718574	0.017022245	0.016718574	0.016718574
		7	6	2	1	3	8	4	5
	**3**	0.018106313	0.018145001	0.018301037	0.01833695	0.018420482	0.018389951	0.018420443	0.018420749
		1	2	3	4	7	5	6	8
	**5**	0.01969191	0.019492778	0.019851822	0.020177351	0.020565273	0.0203605	0.02056417	0.020565123
		2	1	3	4	8	5	6	7
	**7**	0.018679137	0.019673396	0.020023787	0.020581495	0.021456467	0.021345325	0.021436989	0.021449958
		1	2	3	4	8	5	6	7
	**9**	0.018075058	0.018568422	0.020245945	0.020591129	0.022071946	0.021887898	0.022039127	0.022046297
		1	2	3	4	8	5	6	7
	**11**	0.016784194	0.017813806	0.020297419	0.020196387	0.022533239	0.022335852	0.022486436	0.022448058
		1	2	4	3	8	5	7	6
	**15**	0.015435167	0.016319383	0.018953695	0.018602452	0.023138027	0.022918496	0.023029698	0.022898475
		1	2	4	3	8	6	7	5
	**20**	0.012805913	0.013328212	0.016970319	0.015422101	0.02231598	0.022117888	0.022146291	0.02165973
		1	2	4	3	8	6	7	5
	**Average rank**	1.875	2.375	3.25	3.25	7.25	5.625	6.125	6.25
	**Overall rank**	1	2	3	4	8	5	6	7
**PSNR**	**2**	35.49459253	35.49883795	35.53427678	35.54025969	35.53178815	35.37448739	35.53178815	35.53178815
		7	6	2	1	3	8	4	5
	**3**	34.82257761	34.81831091	34.74656267	34.72995823	34.69034264	34.70439468	34.69036781	34.6902191
		1	2	3	4	7	5	6	8
	**5**	34.10868353	34.19383082	34.0356074	33.8997311	33.73461242	33.82121997	33.73508758	33.73467265
		2	1	3	4	8	5	6	7
	**7**	34.51910531	34.11363961	33.95844561	33.7285504	33.36646194	33.41169175	33.37433363	33.36907407
		1	2	3	4	8	5	6	7
	**9**	34.83142146	34.61202481	33.85756398	33.720252	33.12097482	33.19309431	33.13378548	33.13107723
		1	2	3	4	8	5	6	7
	**11**	35.49571095	34.97009784	33.83043	33.89151441	32.94135205	33.0172122	32.95934543	32.97427452
		1	2	4	3	8	5	7	6
	**15**	36.2118861	35.73935676	34.39936665	34.60201521	32.71143402	32.79384831	32.75214232	32.80148627
		1	2	4	3	8	6	7	5
	**20**	37.84334649	37.48833837	35.36955413	36.2383308	33.02542851	33.10236325	33.09165081	33.2838106
		1	2	4	3	8	6	7	5
	**Average rank**	1.875	2.375	3.25	3.25	7.25	5.625	6.125	6.25
	**Overall rank**	1	2	3	4	8	5	6	7
**SSIM**	**2**	0.901954875	0.902269344	0.902464634	0.90259794	0.902494529	0.901093979	0.902494529	0.902494529
		7	6	5	1	2	8	3	4
	**3**	0.902782444	0.902349141	0.901816779	0.901454328	0.90080113	0.901092343	0.900801356	0.900799703
		1	2	3	4	7	5	6	8
	**5**	0.893180718	0.895060895	0.891987476	0.888818567	0.885862137	0.887340624	0.885898374	0.885875571
		2	1	3	4	8	5	6	7
	**7**	0.900573762	0.89333858	0.889126288	0.885036815	0.877545465	0.878243976	0.877710208	0.877612163
		1	2	3	4	8	5	6	7

	**9**	0.906773581	0.90225235	0.887160899	0.884051458	0.871195466	0.872635049	0.8714401	0.871390592
		1	2	3	4	8	5	6	7
	**11**	0.917636717	0.909385592	0.884788067	0.88785291	0.866082313	0.86770084	0.866448568	0.866749876
		1	2	4	3	8	5	7	6
	**15**	0.926788	0.920342191	0.897084906	0.901994729	0.858780001	0.860978667	0.859758975	0.86084299
		1	2	4	3	8	5	7	6
	**20**	0.938045241	0.93553088	0.913308421	0.921763248	0.867807577	0.869882419	0.869557959	0.873912073
		1	2	4	3	8	6	7	5
	**Average rank**	1.875	2.375	3.625	3.25	7.125	5.5	6	6.25
	**Overall rank**	1	2	4	3	8	5	6	7
**FSIM**	**2**	0.478340235	0.480976688	0.472296279	0.477324686	0.474657501	0.505927456	0.474657501	0.474657501
		3	2	8	4	5	1	6	7
	**3**	0.593439925	0.596727027	0.587544456	0.590202517	0.578852876	0.591811777	0.578897714	0.578784004
		2	1	5	4	7	3	6	8
	**5**	0.625137517	0.629851974	0.634297359	0.622473308	0.601265244	0.614770422	0.601922417	0.601161549
		3	2	1	4	7	5	6	8
	**7**	0.643157014	0.630301486	0.622842395	0.621897401	0.603690475	0.622264156	0.607003307	0.605345639
		1	2	3	5	8	4	6	7
	**9**	0.659462717	0.640211869	0.600701799	0.595183958	0.609860362	0.627329379	0.613406672	0.614084072
		1	2	7	8	6	3	5	4
	**11**	0.699185067	0.676388633	0.573300733	0.602832968	0.602757253	0.617107299	0.606748066	0.611448433
		1	2	8	6	7	3	5	4
	**15**	0.731184506	0.713882105	0.62856422	0.659684287	0.58456477	0.589491797	0.590556673	0.589741915
		1	2	4	3	8	7	5	6
	**20**	0.734720047	0.730241988	0.69206787	0.698002521	0.600526075	0.602261293	0.612184937	0.610737434
		1	2	4	3	8	7	5	6
	**Average rank**	1.625	1.875	5	4.625	7	4.125	5.5	6.25
	**Overall rank**	1	2	5	4	8	3	6	7

Table S2. The comparison of statistical results of the values of performance evaluation metrics for all algorithms on test image 'img2'.

Metric	numTh	LSPIPSO	PSO	BFO	GBMO	EMA	MWOA	HWOA	CSO
**RMSE**	**2**	0.016429018	0.016602764	0.016308564	0.016216389	0.016686496	0.018748005	0.016246134	0.016246134
		5	6	4	1	7	8	2	3
	**3**	0.01693909	0.016986103	0.016617539	0.016740733	0.016837165	0.018251809	0.016266157	0.016266114
		6	7	3	4	5	8	2	1
	**5**	0.017889299	0.018526099	0.018321251	0.01846433	0.017707453	0.018316199	0.017289905	0.017290728
		4	8	6	7	3	5	1	2
	**7**	0.018275513	0.01819398	0.019177325	0.018733431	0.018459994	0.018503579	0.017983545	0.017883978
		4	3	8	7	5	6	2	1
	**9**	0.018022163	0.018510309	0.019368639	0.019139994	0.018787978	0.018867576	0.018601996	0.018407315
		1	3	8	7	5	6	4	2
	**11**	0.017731909	0.018044274	0.019344288	0.019059961	0.01917648	0.019162364	0.018894726	0.018772789
		1	2	8	5	7	6	4	3
	**15**	0.016744723	0.017438229	0.019311768	0.01858154	0.019583095	0.019663905	0.019303525	0.01931123

		1	2	6	3	7	8	4	5
	**20**	0.013509943	0.013908033	0.018063931	0.015379968	0.019557594	0.019389268	0.018731474	0.019037254
		1	2	4	3	8	7	5	6
	**Average rank**	2.875	4.125	5.875	4.625	5.875	6.75	3	2.875
	**Overall rank**	1	4	6	5	7	8	3	2
**PSNR**	**2**	35.6689598	35.57048133	35.73889326	35.79063645	35.5215106	34.50261268	35.77535892	35.77535892
		5	6	4	1	7	8	2	3
	**3**	35.34213717	35.3565868	35.5691884	35.47791631	35.43091862	34.71295516	35.7616706	35.76169643
		7	6	3	4	5	8	2	1
	**5**	34.93858168	34.61961508	34.70536448	34.63073065	35.00474252	34.68851163	35.23123143	35.23080998
		4	8	5	7	3	6	1	2
	**7**	34.74273811	34.76100097	34.33224725	34.53014952	34.6368203	34.61121898	34.88401284	34.93659674
		4	3	8	7	5	6	2	1
	**9**	34.86443097	34.62793156	34.23668895	34.3456118	34.4908133	34.45261925	34.581709	34.68156684
		1	3	8	7	5	6	4	2
	**11**	34.98231868	34.83568047	34.25044483	34.37977414	34.31823764	34.33151101	34.4485891	34.51066757
		1	2	8	5	7	6	4	3
	**15**	35.50337168	35.16733917	34.27191896	34.60838109	34.14449838	34.11176011	34.26699295	34.26774343
		1	2	4	3	7	8	6	5
	**20**	37.3217737	37.10730069	34.81199267	36.21524526	34.15663505	34.23546742	34.53030414	34.38986053
		1	2	4	3	8	7	5	6
	**Average rank**	3	4	5.5	4.625	5.875	6.875	3.25	2.875
	**Overall rank**	2	4	6	5	7	8	3	1
**SSIM**	**2**	0.892508179	0.893898246	0.891533899	0.890724185	0.894425544	0.913119678	0.891046658	0.891046658
		4	3	5	8	2	1	6	7
	**3**	0.924712107	0.923767148	0.924201161	0.923724335	0.923558579	0.922778729	0.924221115	0.924221681
		1	5	4	6	7	8	3	2
	**5**	0.931512917	0.92839705	0.929399324	0.928263885	0.931937859	0.928909077	0.934251959	0.934242886
		4	7	5	8	3	6	1	2
	**7**	0.931548313	0.931399266	0.925710322	0.92859266	0.929574867	0.929516694	0.9328173	0.933506956
		3	4	8	7	5	6	2	1
	**9**	0.933831666	0.930909138	0.924210306	0.926479192	0.928242369	0.927886286	0.929619424	0.931165204
		1	3	8	7	5	6	4	2
	**11**	0.935607518	0.933854815	0.924659366	0.92739675	0.925805213	0.926111539	0.928017771	0.929152941
		1	2	8	5	7	6	4	3
	**15**	0.941997836	0.938247114	0.92469267	0.930819134	0.922809077	0.922281718	0.925203845	0.925446213
		1	2	6	3	7	8	5	4
	**20**	0.955624784	0.953886636	0.931341284	0.94670634	0.922846559	0.924210996	0.929173538	0.9271486
		1	2	4	3	8	7	5	6
	**Average rank**	2	3.5	6	5.875	5.5	6	3.75	3.375
	**Overall rank**	1	3	7	6	5	8	4	2
**FSIM**	**2**	0.440960647	0.431415134	0.436985683	0.440606508	0.418039001	0.340682961	0.436539326	0.436539326
		1	6	3	2	7	8	4	5
	**3**	0.5092607	0.514855086	0.541943133	0.522955815	0.508474218	0.429296493	0.545152546	0.545142969
		6	5	3	4	7	8	1	2

	**5**	0.502949379	0.468024287	0.50062951	0.468343927	0.525124658	0.489880383	0.564445513	0.562998331
		4	8	5	7	3	6	1	2
	**7**	0.521983793	0.508943792	0.424855445	0.479836317	0.496840071	0.515530818	0.554388737	0.566035136
		3	5	8	7	6	4	2	1
	**9**	0.532922165	0.507688565	0.374106136	0.434072979	0.496408689	0.503287048	0.522474963	0.560524602
		2	4	8	7	6	5	3	1
	**11**	0.579061835	0.551828551	0.393367803	0.452315123	0.47085791	0.481795083	0.515711166	0.55872537
		1	3	8	7	6	5	4	2
	**15**	0.690899751	0.643701266	0.376347523	0.524234187	0.425220056	0.414083464	0.494501484	0.51772536
		1	2	8	3	6	7	5	4
	**20**	0.712021847	0.704024982	0.44762911	0.612380579	0.392568163	0.416792138	0.53293346	0.489633899
		1	2	6	3	8	7	4	5
	**Average rank**	2.375	4.375	6.125	5	6.125	6.25	3	2.75
	**Overall rank**	1	4	6	5	7	8	3	2

Table S3. The comparison of statistical results of the values of performance evaluation metrics for all algorithms on test image 'img3'.

Metric	numTh	LSPIPSO	PSO	BFO	GBMO	EMA	MWOA	HWOA	CSO
**RMSE**	**2**	0.024372687	0.024160429	0.024414164	0.024429857	0.024432188	0.024413542	0.024432188	0.024432188
		2	1	4	5	6	3	7	8
	**3**	0.024808652	0.02488675	0.024895116	0.024964075	0.025044566	0.024969517	0.025044513	0.025045319
		1	2	3	4	7	5	6	8
	**5**	0.024551547	0.023923472	0.024999919	0.025257677	0.025734145	0.025660025	0.025739002	0.025742386
		2	1	3	4	6	5	7	8
	**7**	0.022133306	0.023431864	0.024698984	0.025023438	0.02603863	0.025926193	0.026024206	0.026036708
		1	2	3	4	8	5	6	7
	**9**	0.020257886	0.021107247	0.024234823	0.024232746	0.026232426	0.026109859	0.026206831	0.026196908
		1	2	4	3	8	5	7	6
	**11**	0.019152286	0.02002218	0.023792844	0.023188529	0.026369261	0.026247636	0.026328252	0.026265329
		1	2	4	3	8	5	7	6
	**15**	0.017317051	0.018115297	0.022177565	0.021099895	0.026441111	0.026367047	0.026441917	0.026244346
		1	2	4	3	7	6	8	5
	**20**	0.013780014	0.014562351	0.018975322	0.016701596	0.026213352	0.026118086	0.026102233	0.025603288
		1	2	4	3	8	7	6	5
	**Average rank**	1.25	1.75	3.625	3.625	7.25	5.125	6.75	6.625
	**Overall rank**	1	2	3	4	8	5	7	6
**PSNR**	**2**	32.25507363	32.32124685	32.24072844	32.23543352	32.23461407	32.24100753	32.23461407	32.23461407
		2	1	4	5	6	3	7	8
	**3**	32.10086186	32.07294793	32.07032986	32.0468719	32.01899775	32.04460261	32.01902123	32.01873191
		1	2	3	4	7	5	6	8
	**5**	32.18907682	32.40733934	32.02994725	31.94341101	31.78223972	31.80680779	31.78055751	31.77940724
		2	1	3	4	6	5	7	8
	**7**	33.09203613	32.59275922	32.13087439	32.02380781	31.67953781	31.71662732	31.68424315	31.68012196
		1	2	3	4	8	5	6	7

	**9**	33.85528237	33.46242714	32.28842601	32.30186273	31.61464481	31.65499951	31.62294365	31.62630447
		1	2	4	3	8	5	7	6
	**11**	34.32640309	33.95503259	32.45447322	32.68516208	31.56905771	31.6086748	31.58247022	31.60308941
		1	2	4	3	8	5	7	6
	**15**	35.22559143	34.81997955	33.05589827	33.50240429	31.54464166	31.56884575	31.54444329	31.60860477
		1	2	4	3	7	6	8	5
	**20**	37.18183118	36.73897535	34.38044803	35.51011134	31.62024035	31.65122673	31.65750046	31.82353707
		1	2	4	3	8	7	6	5
	**Average rank**	1.25	1.75	3.625	3.625	7.25	5.125	6.75	6.625
	**Overall rank**	1	2	3	4	8	5	7	6
**SSIM**	**2**	0.854442051	0.854372117	0.85438397	0.854272532	0.854327479	0.854880924	0.854327479	0.854327479
		2	4	3	8	5	1	6	7
	**3**	0.852463369	0.852009819	0.851944867	0.851212882	0.85040902	0.851171706	0.850406681	0.850403286
		1	2	3	4	6	5	7	8
	**5**	0.856322198	0.86236882	0.851489478	0.848865797	0.844270628	0.845125244	0.844278474	0.844269669
		2	1	3	4	7	5	6	8
	**7**	0.87865291	0.867315641	0.853937333	0.850826961	0.841284283	0.842421388	0.841464422	0.841360639
		1	2	3	4	8	5	6	7
	**9**	0.893331392	0.885552903	0.858345717	0.860082478	0.839288629	0.840330982	0.839575838	0.839729442
		1	2	4	3	8	5	7	6
	**11**	0.900344454	0.894491615	0.863302768	0.870333911	0.837820241	0.838624882	0.838243111	0.838855198
		1	2	4	3	8	6	7	5
	**15**	0.912603487	0.906949924	0.87759859	0.884783485	0.83673609	0.837237236	0.836865674	0.838254639
		1	2	4	3	8	6	7	5
	**20**	0.927359454	0.924034708	0.898587623	0.911060728	0.838792408	0.839674305	0.839984642	0.844295113
		1	2	4	3	8	7	6	5
	**Average rank**	1.25	2.125	3.5	4	7.25	5	6.5	6.375
	**Overall rank**	1	2	3	4	8	5	7	6
**FSIM**	**2**	0.373888202	0.36748598	0.37102	0.36936394	0.369391913	0.37303241	0.369391913	0.369391913
		1	8	3	7	4	2	5	6
	**3**	0.404446513	0.400362225	0.422996121	0.405739601	0.402119624	0.392310612	0.402092986	0.402090185
		3	7	1	2	4	8	5	6
	**5**	0.51833911	0.468804562	0.435897143	0.426685361	0.405669347	0.402544716	0.406203218	0.40673829
		1	2	3	4	7	8	6	5
	**7**	0.61570014	0.557195114	0.436098967	0.418552465	0.40139682	0.397703096	0.403423311	0.402223872
		1	2	3	4	7	8	5	6
	**9**	0.665206828	0.625621157	0.474952997	0.511728992	0.394300339	0.384500908	0.398320085	0.399649917
		1	2	4	3	7	8	6	5
	**11**	0.676336454	0.661177534	0.526081881	0.586237676	0.382491429	0.360956946	0.390201286	0.389517372
		1	2	4	3	7	8	5	6
	**15**	0.703080952	0.694378787	0.618182466	0.632983059	0.369688793	0.363285724	0.382266401	0.368362727
		1	2	4	3	6	8	5	7
	**20**	0.69613299	0.691124462	0.676676401	0.656509205	0.370907375	0.380134008	0.393232035	0.412253427
		1	2	3	4	8	7	6	5
	**Average rank**	1.25	3.375	3.125	3.75	6.25	7.125	5.375	5.75

	Overall rank	1	3	2	4	7	8	5	6

Table S4. The comparison of statistical results of the values of performance evaluation metrics for all algorithms on test image 'img4'.

Metric	numTh	LSPIPSO	PSO	BFO	GBMO	EMA	MWOA	HWOA	CSO
RMSE	2	0.016584347	0.016397383	0.016397341	0.016397948	0.01639732	0.017494026	0.01639732	0.01639732
		7	5	4	6	1	8	2	3
	3	0.018218925	0.018218076	0.018178257	0.01821555	0.018178297	0.018954458	0.018178302	0.018178297
		7	6	1	5	2	8	4	3
	5	0.022067897	0.022032662	0.021969793	0.022028921	0.02203456	0.022215404	0.022034582	0.022034602
		7	3	1	2	4	8	5	6
	7	0.022965071	0.023605164	0.023741951	0.02362929	0.023714056	0.023774619	0.023714055	0.023714219
		1	2	7	3	5	8	4	6
	9	0.023880256	0.023354485	0.024947134	0.024558975	0.024899837	0.024945816	0.024900229	0.024900392
		2	1	8	3	4	7	5	6
	11	0.022236203	0.021969475	0.025827849	0.025061138	0.025818705	0.025818057	0.025816049	0.025824506
		2	1	8	3	6	5	4	7
	15	0.020430789	0.019967941	0.02712576	0.022358457	0.027104003	0.027048528	0.02708255	0.027083053
		2	1	8	3	7	4	5	6
	20	0.017795296	0.017819681	0.025638307	0.019846374	0.025626918	0.025547794	0.025554731	0.025532782
		1	2	8	3	7	5	6	4
	Average rank	3.625	2.625	5.625	3.5	4.5	6.625	4.375	5.125
	Overall rank	3	1	7	2	5	8	4	6
PSNR	2	35.4921558	35.58461497	35.58463745	35.5843431	35.58464704	35.0263139	35.58464704	35.58464704
		7	5	4	6	1	8	2	3
	3	34.59321331	34.5974419	34.60882117	34.61079457	34.60878763	34.28559308	34.6087845	34.60878763
		7	6	2	1	3	8	5	4
	5	32.96690999	32.98464823	32.99955915	32.99905911	32.97711953	32.91753778	32.97711286	32.97710436
		7	3	1	2	4	8	5	6
	7	32.6242741	32.40418998	32.35017994	32.4002241	32.35838537	32.34545976	32.35838118	32.35832471
		1	2	7	3	4	8	5	6
	9	32.33063374	32.50691892	31.93388318	32.07454418	31.94764942	31.93680482	31.94751943	31.94747511
		2	1	8	3	4	7	5	6
	11	32.94478328	33.04602411	31.6361891	31.89520545	31.64161609	31.64476309	31.64226303	31.6399523
		2	1	8	3	6	4	5	7
	15	33.67405936	33.86019282	31.22769609	32.89456107	31.23298893	31.25181635	31.23837044	31.23855669
		2	1	8	3	7	4	6	5
	20	34.78315923	34.7482	31.71943409	33.86457957	31.7108682	31.7359005	31.72970824	31.73941411
		1	2	7	3	8	5	6	4
	Average rank	3.625	2.625	5.625	3	4.625	6.5	4.875	5.125
	Overall rank	3	1	7	2	4	8	5	6
SSIM	2	0.892979643	0.893605537	0.893606736	0.893587392	0.893607388	0.876399411	0.893607388	0.893607388
		7	5	4	6	1	8	2	3
	3	0.892239064	0.892309521	0.892635021	0.892537679	0.892649876	0.882977729	0.892649749	0.892649876

		7	6	4	5	1	8	3	2
	**5**	0.867865871	0.869142525	0.870163369	0.869528499	0.869864653	0.867932484	0.869864234	0.86986438
		8	6	1	5	2	7	4	3
	**7**	0.860021738	0.856354553	0.85832401	0.857620414	0.858787913	0.858231918	0.858787918	0.858788324
		1	8	5	7	4	6	3	2
	**9**	0.851710752	0.851999082	0.849882469	0.848355016	0.85068243	0.850302433	0.850680494	0.850687281
		2	1	7	8	4	6	5	3
	**11**	0.856975284	0.859403074	0.843413674	0.841939665	0.844226562	0.844177627	0.844240501	0.844204611
		2	1	7	8	4	6	3	5
	**15**	0.860785784	0.861794193	0.834112048	0.853388369	0.834967093	0.835345948	0.83513996	0.835056391
		2	1	8	3	7	4	5	6
	**20**	0.854305731	0.854851926	0.843149064	0.851856567	0.844942865	0.845608687	0.845789302	0.844754254
		2	1	8	3	6	5	4	7
	**Average rank**	3.875	3.625	5.5	5.625	3.625	6.25	3.625	3.875
	**Overall rank**	4	1	6	7	2	8	3	5
**FSIM**	**2**	0.333980574	0.334489728	0.334465998	0.334700093	0.334434945	0.35166029	0.334434945	0.334434945
		8	3	4	2	5	1	6	7
	**3**	0.375281291	0.375034979	0.373073723	0.374175565	0.37238103	0.404360222	0.37238335	0.37238103
		2	3	5	4	7	1	6	8
	**5**	0.400424131	0.400285748	0.381354957	0.394405008	0.374212531	0.390126862	0.374198943	0.374165563
		1	2	5	3	6	4	7	8
	**7**	0.432935258	0.439218328	0.390175708	0.421332294	0.376438232	0.387864799	0.376329172	0.376117837
		2	1	4	3	6	5	7	8
	**9**	0.456052497	0.450314502	0.404448973	0.446623528	0.382584032	0.397364034	0.382074327	0.381309263
		1	2	4	3	6	5	7	8
	**11**	0.451396602	0.457061443	0.416310744	0.435967097	0.388057787	0.407463436	0.387059501	0.385601615
		2	1	4	3	6	5	7	8
	**15**	0.436752045	0.435673224	0.434335955	0.429208369	0.403627148	0.425482394	0.398150712	0.404221705
		1	2	3	4	7	5	8	6
	**20**	0.38557868	0.382625993	0.425242795	0.378555876	0.411398707	0.419980713	0.395759882	0.422151713
		6	7	1	8	4	3	5	2
	**Average rank**	2.875	2.625	3.75	3.75	5.875	3.625	6.625	6.875
	**Overall rank**	2	1	4	5	6	3	7	8

Table S5. The comparison of statistical results of the values of performance evaluation metrics for all algorithms on test image 'img5'.

Metric	numTh	LSPIPSO	PSO	BFO	GBMO	EMA	MWOA	HWOA	CSO
**RMSE**	**2**	0.024017024	0.024192159	0.024018056	0.023993582	0.024017571	0.028817885	0.024017571	0.024017571
		2	7	6	1	3	8	4	5
	**3**	0.025559141	0.024940948	0.025026688	0.024952236	0.025179654	0.027254527	0.025179638	0.025179684
		7	1	3	2	5	8	4	6
	**5**	0.02813244	0.027550898	0.026708413	0.027697768	0.027140678	0.027096566	0.027140553	0.027140786
		8	6	1	7	4	2	3	5
	**7**	0.026149235	0.028613604	0.027837354	0.029028254	0.028007522	0.027893752	0.027997158	0.027998475

		1	7	2	8	6	3	4	5
	**9**	0.026860867	0.027738167	0.029063399	0.029868698	0.028644987	0.028655889	0.028604516	0.028605005
		1	2	7	8	5	6	3	4
	**11**	0.026703319	0.026844257	0.029674574	0.029095057	0.029240383	0.029252504	0.029076083	0.029055445
		1	2	8	5	6	7	4	3
	**15**	0.025165562	0.026200626	0.030424112	0.0281598	0.030152895	0.030053556	0.029723875	0.029675943
		1	2	8	3	7	6	5	4
	**20**	0.019868086	0.020927742	0.030120304	0.022762017	0.029716182	0.029634473	0.029520594	0.029114824
		1	2	8	3	7	6	5	4
	**Average rank**	2.75	3.625	5.375	4.625	5.375	5.75	4	4.5
	**Overall rank**	1	2	6	5	7	8	3	4
**PSNR**	**2**	32.23977605	32.18375228	32.23936174	32.24707105	32.23957696	30.76301622	32.23957696	32.23957696
		2	7	6	1	3	8	4	5
	**3**	31.7696711	31.9435461	31.94340382	31.94954074	31.88301315	31.24293612	31.88301766	31.88300447
		7	2	3	1	5	8	4	6
	**5**	30.99182606	31.17311091	31.43990746	31.1212564	31.29071634	31.30305752	31.2907533	31.29067885
		8	6	1	7	4	2	3	5
	**7**	31.57967057	30.85900983	31.08526028	30.73698065	31.03317329	31.06532783	31.03612961	31.03573644
		1	7	2	8	6	3	4	5
	**9**	31.40184982	31.09182881	30.72811753	30.48716791	30.84521363	30.84256637	30.85715176	30.85700903
		1	2	7	8	5	6	3	4
	**11**	31.45929364	31.39898707	30.54524321	30.70974401	30.6723397	30.66951172	30.71929313	30.72523929
		1	2	8	5	6	7	4	3
	**15**	31.96422006	31.62382333	30.33310881	30.9982552	30.41090438	30.43886793	30.53159781	30.54543942
		1	2	8	3	7	6	5	4
	**20**	33.98152915	33.55995509	30.40590919	32.84431996	30.53646726	30.56003718	30.59234434	30.7080754
		1	2	8	3	7	6	5	4
	**Average rank**	2.75	3.75	5.375	4.5	5.375	5.75	4	4.5
	**Overall rank**	1	2	6	4	7	8	3	5
**SSIM**	**2**	0.797039102	0.797243095	0.797012439	0.797273234	0.797052529	0.801633136	0.797052529	0.797052529
		7	3	8	2	4	1	5	6
	**3**	0.819545578	0.823349679	0.82134338	0.822993657	0.820922268	0.814505755	0.820922501	0.820921936
		7	1	3	2	5	8	4	6
	**5**	0.818813189	0.822527179	0.826640142	0.82175105	0.824267181	0.824702507	0.824268403	0.824266649
		8	6	1	7	4	2	3	5
	**7**	0.834408688	0.818053203	0.823342182	0.815183658	0.821750787	0.82274473	0.8218301	0.821822002
		1	7	2	8	6	3	4	5
	**9**	0.832414576	0.825449896	0.815928389	0.810206001	0.818879158	0.818917258	0.819118174	0.819118586
		1	2	7	8	6	5	4	3
	**11**	0.834167388	0.833147488	0.811974767	0.816389013	0.815326886	0.815320072	0.81652441	0.816698269
		1	2	8	5	6	7	4	3
	**15**	0.84510023	0.837613448	0.806744541	0.823266862	0.808982714	0.809823627	0.812339392	0.812724713
		1	2	8	3	7	6	5	4
	**20**	0.875332835	0.870784342	0.80832089	0.862367651	0.811647143	0.812323709	0.813089217	0.816061544
		1	2	8	3	7	6	5	4

	**Average rank**	3.375	3.125	5.625	4.75	5.625	4.75	4.25	4.5
	**Overall rank**	2	1	7	5	8	6	3	4
**FSIM**	**2**	0.347662582	0.34741057	0.347910722	0.347788255	0.344993661	0.340971792	0.344993661	0.344993661
		3	4	1	2	5	8	6	7
	**3**	0.418389304	0.41941849	0.410379507	0.416640213	0.403967035	0.403528649	0.403987477	0.403945289
		2	1	4	3	6	8	5	7
	**5**	0.435439337	0.440735833	0.43853009	0.430073862	0.407548893	0.43504554	0.407701475	0.407526737
		3	1	2	5	7	4	6	8
	**7**	0.529157307	0.46685762	0.444437475	0.43277806	0.41497344	0.440674951	0.414549707	0.412595492
		1	2	3	5	6	4	7	8
	**9**	0.578898121	0.508986117	0.451569837	0.41285632	0.421237779	0.446816109	0.418725152	0.419211186
		1	2	3	8	5	4	7	6
	**11**	0.557781891	0.563883779	0.446581685	0.47956586	0.418513865	0.441456196	0.419340142	0.424580452
		2	1	4	3	8	5	7	6
	**15**	0.617245771	0.601804042	0.416572863	0.522343945	0.402633358	0.432335475	0.42177621	0.444610891
		1	2	7	3	8	5	6	4
	**20**	0.594624261	0.600100765	0.385256448	0.519595716	0.411768904	0.4186169	0.406353228	0.449105243
		2	1	8	3	6	5	7	4
	**Average rank**	1.875	1.75	4	4	6.375	5.375	6.375	6.25
	**Overall rank**	2	1	3	4	7	5	8	6

Table S6. The comparison of statistical results of the values of performance evaluation metrics for all algorithms on test image 'img6'.

**Metric**	**numTh**	**LSPIPSO**	**PSO**	**BFO**	**GBMO**	**EMA**	**MWOA**	**HWOA**	**CSO**
**RMSE**	**2**	0.025460913	0.025467803	0.02530241	0.025460913	0.025309881	0.026948939	0.025309881	0.025309881
		5	7	1	6	2	8	3	4
	**3**	0.022425932	0.022274877	0.022633731	0.022425932	0.022575117	0.024993355	0.022575342	0.022509536
		2	1	7	3	5	8	6	4
	**5**	0.020144937	0.020665149	0.022084671	0.020144937	0.020868991	0.024511566	0.020549421	0.020555524
		1	5	7	2	6	8	3	4
	**7**	0.018776438	0.018841398	0.020471139	0.018776438	0.019619113	0.022982808	0.018680985	0.018486174
		3	5	7	4	6	8	2	1
	**9**	0.017373033	0.018178494	0.019948371	0.017373033	0.018585747	0.023239166	0.018157672	0.017793894
		1	5	7	2	6	8	4	3
	**11**	0.017407984	0.01837524	0.019983658	0.017407984	0.018523107	0.022147445	0.018237596	0.017677619
		1	5	7	2	6	8	4	3
	**15**	0.017150065	0.018104973	0.020309723	0.017150065	0.018210084	0.022772966	0.018835165	0.018023028
		1	4	7	2	5	8	6	3
	**20**	0.016749279	0.017133654	0.019091373	0.016749279	0.016859062	0.020880869	0.01788813	0.017182503
		1	4	7	2	3	8	6	5
	**Average rank**	1.875	4.5	6.25	2.875	4.875	8	4.25	3.375
	**Overall rank**	1	5	7	2	6	8	4	3
**PSNR**	**2**	31.88102756	31.87701021	31.92834991	31.88102756	31.93322068	31.38459969	31.93322068	31.93322068
		5	7	4	6	1	8	2	3

	**3**	32.98543194	33.01916447	32.87680125	32.98543194	32.92498167	32.04147795	32.92488931	32.94969382
		2	1	7	3	5	8	6	4
	**5**	33.89480683	33.65778093	33.09606393	33.89480683	33.608816	32.20961211	33.74324679	33.7405761
		1	5	7	2	6	8	3	4
	**7**	34.48498264	34.46673586	33.7261272	34.48498264	34.14505984	32.73671813	34.57033117	34.66133024
		3	5	7	4	6	8	2	1
	**9**	35.17393222	34.78087689	33.96898486	35.17393222	34.61093046	32.63176129	34.81795996	34.9898916
		1	5	7	2	6	8	4	3
	**11**	35.16162319	34.68460378	33.97033221	35.16162319	34.64165745	33.06995951	34.77913865	35.05063851
		1	5	7	2	6	8	4	3
	**15**	35.315984	34.83395056	33.82936153	35.315984	34.78398358	32.83828051	34.49913626	34.8819562
		1	4	7	2	5	8	6	3
	**20**	35.49787962	35.30377865	34.37561172	35.49787962	35.46307418	33.59365016	34.94655097	35.29426492
		1	4	7	2	3	8	6	5
	**Average rank**	1.875	4.5	6.625	2.875	4.75	8	4.125	3.25
	**Overall rank**	1	5	7	2	6	8	4	3
**SSIM**	**2**	0.79277931	0.79300586	0.794192311	0.79277931	0.793177662	0.790689459	0.793177662	0.793177662
		6	5	1	7	2	8	3	4
	**3**	0.833640267	0.835601788	0.831758398	0.833640267	0.832455307	0.81526813	0.832458578	0.832880616
		2	1	7	3	6	8	5	4
	**5**	0.860895799	0.857838002	0.84832527	0.860895799	0.857369222	0.832857188	0.859089511	0.858699977
		1	5	7	2	6	8	3	4
	**7**	0.872376703	0.871301047	0.859429718	0.872376703	0.867612034	0.844447134	0.872778208	0.872787429
		3	5	7	4	6	8	2	1
	**9**	0.879459486	0.874909921	0.863149466	0.879459486	0.872091714	0.842753017	0.873610444	0.875286702
		1	4	7	2	6	8	5	3
	**11**	0.876936393	0.871545679	0.861456634	0.876936393	0.870661124	0.849497006	0.870272394	0.872285685
		1	4	7	2	5	8	6	3
	**15**	0.868639504	0.867770648	0.854282881	0.868639504	0.866190665	0.841633193	0.859504605	0.86362641
		1	3	7	2	4	8	6	5
	**20**	0.857420208	0.858288693	0.849320919	0.857420208	0.860744829	0.845935033	0.848674601	0.852796487
		3	2	6	4	1	8	7	5
	**Average rank**	2.25	3.625	6.125	3.25	4.5	8	4.625	3.625
	**Overall rank**	1	3	7	2	5	8	6	4
**FSIM**	**2**	0.594637668	0.595900398	0.596980098	0.593119308	0.591282606	0.626108918	0.591282606	0.591282606
		4	3	2	5	6	1	7	8
	**3**	0.661241177	0.663211903	0.662197658	0.657939774	0.656861758	0.666381827	0.65689989	0.656909463
		4	2	3	5	8	1	7	6
	**5**	0.722467098	0.714961079	0.709936696	0.714906994	0.71097196	0.739631999	0.709020376	0.707784751
		2	3	6	4	5	1	7	8
	**7**	0.739377898	0.730697653	0.726718	0.734814966	0.728721136	0.754712537	0.72223883	0.719469604
		2	4	6	3	5	1	7	8
	**9**	0.760020587	0.749869394	0.740903571	0.753008896	0.738826576	0.787021527	0.731827867	0.733089918
		2	4	5	3	6	1	8	7
	**11**	0.760033575	0.761074555	0.765984985	0.759663409	0.759347352	0.786145797	0.745571327	0.739447275

		4	3	2	5	6	1	7	8
	**15**	0.768182898	0.771595457	0.775320452	0.757686025	0.771669382	0.793510732	0.757930458	0.75418835
		5	4	2	7	3	1	6	8
	**20**	0.776631942	0.766637647	0.772888875	0.761285082	0.782813579	0.803360122	0.755708724	0.755486885
		3	5	4	6	2	1	7	8
	**Average rank**	3.25	3.5	3.75	4.75	5.125	1	7	7.625
	**Overall rank**	2	3	4	5	6	1	7	8

Table S7. The comparison of statistical results of the values of performance evaluation metrics for all algorithms on test image 'img7'.

Metric	numTh	LSPIPSO	PSO	BFO	GBMO	EMA	MWOA	HWOA	CSO
**RMSE**	**2**	0.020290265	0.020326445	0.020033807	0.019987266	0.019840328	0.021891082	0.019840328	0.019840328
		6	7	5	4	1	8	2	3
	**3**	0.016359847	0.016764001	0.017192559	0.016497359	0.016258381	0.01941457	0.016219166	0.016228056
		4	6	7	5	3	8	1	2
	**5**	0.013608977	0.013835681	0.015832259	0.013819768	0.014150035	0.01786106	0.013717155	0.013572594
		2	5	7	4	6	8	3	1
	**7**	0.012098441	0.012557186	0.014412853	0.012867397	0.013486743	0.017366534	0.012874757	0.012572704
		1	2	7	4	6	8	5	3
	**9**	0.012008845	0.012205799	0.014361617	0.012646565	0.013071592	0.015593932	0.012900004	0.012479661
		1	2	7	4	6	8	5	3
	**11**	0.012341538	0.012206653	0.014379108	0.012453919	0.012659467	0.016865441	0.0131596	0.012459396
		2	1	7	3	5	8	6	4
	**15**	0.012886893	0.012729966	0.014958671	0.012760803	0.0128965	0.017301814	0.013977182	0.013000422
		3	1	7	2	4	8	6	5
	**20**	0.01252788	0.012637567	0.0142836	0.012431864	0.012301033	0.016164538	0.01383482	0.012847666
		3	4	7	2	1	8	6	5
	**Average rank**	2.75	3.5	6.75	3.5	4	8	4.25	3.25
	**Overall rank**	1	3	7	4	5	8	6	2
**PSNR**	**2**	33.84160257	33.81777336	33.95062714	33.97641121	34.04443664	33.17256782	34.04443664	34.04443664
		6	7	5	4	1	8	2	3
	**3**	35.65550433	35.48473552	35.25882297	35.63668386	35.76300683	34.19765954	35.78539182	35.78038823
		4	6	7	5	3	8	1	2
	**5**	37.20173408	37.05468891	35.97843695	37.13933391	36.95783844	34.87008699	37.23334832	37.3213053
		3	5	7	4	6	8	2	1
	**7**	38.22748821	37.91272711	36.7866228	37.75136702	37.36318051	35.1382495	37.78385424	37.99345783
		1	3	7	5	6	8	4	2
	**9**	38.34528067	38.23759362	36.78254581	37.90335318	37.64640852	36.08068893	37.76228626	38.05523054
		1	2	7	4	6	8	5	3
	**11**	38.11225069	38.2223901	36.80130198	38.06215427	37.92438824	35.40191559	37.59149714	38.06071341
		2	1	7	3	5	8	6	4
	**15**	37.77616422	37.87150633	36.47962686	37.84265205	37.75533415	35.1940739	37.06650448	37.6957091
		3	1	7	2	4	8	6	5
	**20**	38.02768436	37.94006733	36.87753024	38.07635638	38.18289071	35.80065433	37.16523602	37.80829416

		3	4	7	2	1	8	6	5
	**Average rank**	2.875	3.625	6.75	3.625	4	8	4	3.125
	**Overall rank**	1	3	7	4	5	8	6	2
**SSIM**	**2**	0.830374539	0.830374539	0.830643458	0.830923338	0.831741964	0.822337408	0.831741964	0.831741964
		6	7	5	4	1	8	2	3
	**3**	0.874701764	0.874701764	0.872995844	0.875331549	0.877310297	0.86368574	0.877496403	0.877352103
		5	6	7	4	3	8	1	2
	**5**	0.906780405	0.906780405	0.893994455	0.906670164	0.903206171	0.88423613	0.904762024	0.904448371
		1	2	7	3	6	8	4	5
	**7**	0.914909002	0.914909002	0.904519786	0.911842149	0.910695781	0.892828942	0.912621855	0.911699563
		1	2	7	4	6	8	3	5
	**9**	0.915172466	0.915172466	0.908040824	0.909445294	0.912429286	0.901205763	0.911602517	0.910668419
		1	2	7	6	3	8	4	5
	**11**	0.91308115	0.91308115	0.905445817	0.907776445	0.914009679	0.895338534	0.907061533	0.908836033
		2	3	7	5	1	8	6	4
	**15**	0.908243647	0.908243647	0.898213166	0.90500703	0.90604198	0.889332406	0.895786167	0.89720128
		1	2	5	4	3	8	7	6
	**20**	0.893605919	0.893605919	0.891068331	0.887606003	0.895557556	0.885338817	0.881512941	0.885096169
		2	3	4	5	1	6	8	7
	**Average rank**	2.375	3.375	6.125	4.375	3	7.75	4.375	4.625
	**Overall rank**	1	3	7	4	2	8	5	6
**FSIM**	**2**	0.625781462	0.627598849	0.629166856	0.627266607	0.629283607	0.625781462	0.629283607	0.629283607
		7	5	4	6	1	8	2	3
	**3**	0.714702621	0.710397187	0.7103632	0.703559107	0.704482308	0.714702621	0.704563692	0.704313603
		1	3	4	8	6	2	5	7
	**5**	0.768384036	0.768648419	0.75431115	0.769500721	0.758522984	0.768384036	0.759702012	0.757993153
		3	2	8	1	6	4	5	7
	**7**	0.817390999	0.801355168	0.788565755	0.793618923	0.791827375	0.817390999	0.792645684	0.788679662
		1	3	8	4	6	2	5	7
	**9**	0.820128641	0.814272675	0.820167187	0.800065044	0.814218235	0.820128641	0.8076116	0.801815099
		2	4	1	8	5	3	6	7
	**11**	0.835427061	0.824311185	0.828666484	0.814336732	0.840373078	0.835427061	0.817889825	0.81718035
		2	5	4	8	1	3	6	7
	**15**	0.854312101	0.849423903	0.84605667	0.841535026	0.848737529	0.854312101	0.82810998	0.820835781
		1	3	5	6	4	2	7	8
	**20**	0.857858548	0.851113266	0.854247582	0.842242631	0.858178625	0.857858548	0.833333072	0.838560984
		2	5	4	6	1	3	8	7
	**Average rank**	2.375	3.75	4.75	5.875	3.75	3.375	5.5	6.625
	**Overall rank**	1	3	5	7	4	2	6	8

Table S8. The comparison of statistical results of the values of performance evaluation metrics for all algorithms on test image 'img8'.

Metric	numTh	LSPIPSO	PSO	BFO	GBMO	EMA	MWOA	HWOA	CSO
**RMSE**	**2**	0.018696455	0.018683008	0.018750267	0.018679024	0.018696455	0.019688074	0.018696455	0.018696455

		3	2	7	1	4	8	5	6
	**3**	0.015668056	0.016057926	0.016254087	0.015799795	0.015667894	0.017638663	0.015668056	0.015667886
		3	6	7	5	2	8	4	1
	**5**	0.013280646	0.014169901	0.014347759	0.013580964	0.01355715	0.016702429	0.013280646	0.013263571
		2	6	7	5	4	8	3	1
	**7**	0.011891368	0.01319448	0.013176381	0.012104544	0.012584537	0.01674514	0.011891368	0.011907658
		1	7	6	4	5	8	2	3
	**9**	0.011488439	0.012163304	0.01321638	0.01188952	0.012381237	0.01682685	0.011488439	0.01153238
		1	5	7	4	6	8	2	3
	**11**	0.011173973	0.011722994	0.012920974	0.011525511	0.012367663	0.016847695	0.011173973	0.011436857
		1	5	7	4	6	8	2	3
	**15**	0.01160094	0.011999476	0.013282038	0.011510398	0.01221664	0.016464762	0.01160094	0.011582694
		3	5	7	1	6	8	4	2
	**20**	0.010735511	0.010910103	0.011493803	0.010365784	0.01068264	0.015174024	0.010735511	0.010783772
		3	6	7	1	2	8	4	5
	**Average rank**	2.125	5.25	6.875	3.125	4.375	8	3.25	3
	**Overall rank**	1	6	7	3	5	8	4	2
**PSNR**	**2**	34.56268068	34.56874783	34.53687493	34.57015999	34.56268068	34.11032418	34.56268068	34.56268068
		3	2	7	1	4	8	5	6
	**3**	36.09663035	35.87587104	35.76566822	36.02063244	36.09671894	35.06082209	36.09663035	36.09672329
		3	6	7	5	2	8	4	1
	**5**	37.53222266	36.93942252	36.80418782	37.31748401	37.35277388	35.50906457	37.53222266	37.54257255
		2	6	7	5	4	8	3	1
	**7**	38.49257391	37.52684715	37.55642671	38.33301854	37.99909306	35.48089331	38.49257391	38.47905953
		1	7	6	4	5	8	2	3
	**9**	38.78940193	38.23576133	37.51972856	38.45590083	38.13793387	35.45503421	38.78940193	38.75219254
		1	5	7	4	6	8	2	3
	**11**	39.02942712	38.58087667	37.73061419	38.73412487	38.14682964	35.45035985	39.02942712	38.81983331
		1	5	7	4	6	8	2	3
	**15**	38.70552318	38.38471133	37.51309741	38.73536671	38.24746519	35.62446538	38.70552318	38.71580466
		3	5	7	1	6	8	4	2
	**20**	39.37892518	39.22824212	38.75689555	39.67346881	39.41935026	36.34455	39.37892518	39.33905598
		3	6	7	1	2	8	4	5
	**Average rank**	2.125	5.25	6.875	3.125	4.375	8	3.25	3
	**Overall rank**	1	6	7	3	5	8	4	2
**SSIM**	**2**	0.895539208	0.895298919	0.895771776	0.895268149	0.895539208	0.900469673	0.895539208	0.895539208
		3	7	2	8	4	1	5	6
	**3**	0.913853089	0.913450701	0.913395834	0.913978834	0.913853089	0.909533786	0.913837947	0.913835774
		2	6	7	1	3	8	4	5
	**5**	0.93771029	0.934946714	0.933394157	0.937062522	0.93771029	0.924746047	0.938048753	0.937884739
		3	6	7	5	4	8	1	2
	**7**	0.944847529	0.940631326	0.939118723	0.943728058	0.944847529	0.928391046	0.945431242	0.944974936
		3	6	7	5	4	8	1	2
	**9**	0.94715817	0.946227162	0.942719194	0.946984521	0.94715817	0.927807233	0.947779018	0.947174626
		3	6	7	5	4	8	1	2

	11	0.94750569	0.94743763	0.941571292	0.94656075	0.94750569	0.929258058	0.947554893	0.94658141
		2	4	7	6	3	8	1	5
	15	0.945759883	0.945954266	0.942964705	0.945684524	0.945759883	0.930476387	0.943744339	0.943885446
		2	1	7	4	3	8	6	5
	20	0.943567086	0.941259002	0.941605175	0.937036407	0.943567086	0.93450436	0.937582809	0.93697181
		1	4	3	6	2	8	5	7
	Average rank	2.375	5	5.875	5	3.375	7.125	3	4.25
	Overall rank	1	5	7	6	3	8	2	4
FSIM	2	0.747321939	0.739950535	0.743518552	0.737665552	0.737458587	0.747321939	0.737458587	0.737458587
		1	4	3	5	6	2	7	8
	3	0.802113897	0.807146034	0.802363455	0.803056431	0.800596407	0.802113897	0.800496557	0.800490007
		4	1	3	2	6	5	7	8
	5	0.851736143	0.851029614	0.844979054	0.847713143	0.851525277	0.851736143	0.847094718	0.845552656
		1	4	8	5	3	2	6	7
	7	0.8876486	0.862687534	0.858042318	0.857006121	0.86529505	0.8876486	0.856045884	0.853147119
		1	4	5	6	3	2	7	8
	9	0.902549857	0.873714697	0.882080936	0.869753674	0.881415835	0.902549857	0.867830595	0.865594569
		1	5	3	6	4	2	7	8
	11	0.907277864	0.875633892	0.876158005	0.871533823	0.890365735	0.907277864	0.86802769	0.867742696
		1	5	4	6	3	2	7	8
	15	0.918095326	0.892997986	0.904171908	0.881528786	0.89394657	0.918095326	0.877551642	0.877288494
		1	5	3	6	4	2	7	8
	20	0.921508712	0.887427002	0.894191483	0.876107126	0.894449738	0.921508712	0.875697798	0.873562744
		1	5	4	6	3	2	7	8
	Average rank	1.375	4.125	4.125	5.25	4	2.375	6.875	7.875
	Overall rank	1	4	5	6	3	2	7	8

Table S9. The comparison of statistical results of the values of performance evaluation metrics for all algorithms on test image 'img9'.

Metric	numTh	LSPIPSO	PSO	BFO	GBMO	EMA	MWOA	HWOA	CSO
RMSE	2	0.012523969	0.012580359	0.012565954	0.012489454	0.012523969	0.013603775	0.012523969	0.012523969
		2	7	6	1	3	8	4	5
	3	0.010002386	0.010321093	0.010472632	0.009820622	0.010002386	0.011804513	0.010021189	0.010014366
		2	6	7	1	3	8	5	4
	5	0.00778073	0.008401489	0.00900461	0.007852414	0.00778073	0.010199746	0.007757245	0.007786946
		2	6	7	5	3	8	1	4
	7	0.006946232	0.007348825	0.008031248	0.007449366	0.006946232	0.009951484	0.006930657	0.00691602
		3	5	7	6	4	8	2	1
	9	0.006778618	0.007326994	0.007661929	0.00710341	0.006778618	0.010253108	0.006830094	0.006851952
		1	6	7	5	2	8	3	4
	11	0.006768297	0.007335163	0.007650889	0.006904975	0.006768297	0.010315507	0.006990718	0.007005463
		1	6	7	3	2	8	4	5
	15	0.007038882	0.007373676	0.007952684	0.007518428	0.007038882	0.010114091	0.007397281	0.007290429
		1	4	7	6	2	8	5	3

	**20**	0.0074354	0.00771393	0.008033833	0.008116594	0.0074354	0.009696092	0.008165638	0.007906629
		1	3	5	6	2	8	7	4
	**Average rank**	1.625	5.375	6.625	4.125	2.625	8	3.875	3.75
	**Overall rank**	1	6	7	5	2	8	4	3
**PSNR**	**2**	37.90513447	37.86976237	37.88759232	37.92928421	37.90513447	37.17637307	37.90513447	37.90513447
		2	7	6	1	3	8	4	5
	**3**	39.78347485	39.53467317	39.33621029	39.95321616	39.78347485	38.30391263	39.76793734	39.77449681
		2	6	7	1	3	8	5	4
	**5**	41.91596063	41.25183925	40.58516705	41.87296324	41.91596063	39.52299166	41.98029668	41.94357916
		3	6	7	5	4	8	1	2
	**7**	42.8767697	42.42342477	41.60519969	42.30653255	42.8767697	39.74283316	42.95830024	42.99330767
		3	5	7	6	4	8	2	1
	**9**	43.10287639	42.44119044	41.96274544	42.76461691	43.10287639	39.37705988	43.10976809	43.09329872
		2	6	7	5	3	8	1	4
	**11**	43.15391839	42.51181945	42.04519347	43.01573576	43.15391839	39.37786893	42.95054926	42.91896561
		1	6	7	3	2	8	4	5
	**15**	42.84054631	42.46368517	41.7267575	42.29596989	42.84054631	39.66368296	42.47014329	42.5754184
		1	5	7	6	2	8	4	3
	**20**	42.50925004	42.18108618	41.78623133	41.78359262	42.50925004	40.12518207	41.72195329	42.00248112
		1	3	5	6	2	8	7	4
	**Average rank**	1.875	5.5	6.625	4.125	2.875	8	3.5	3.5
	**Overall rank**	1	6	7	5	2	8	3	4
**SSIM**	**2**	0.899122502	0.89903569	0.898853968	0.899096568	0.899122502	0.903263821	0.899122502	0.899122502
		2	7	8	6	3	1	4	5
	**3**	0.943553456	0.938328039	0.940338492	0.943299093	0.943553456	0.933820179	0.94359472	0.943622205
		3	7	6	5	4	8	2	1
	**5**	0.960094141	0.956825005	0.955429969	0.958950642	0.960094141	0.952301313	0.959696063	0.959286643
		1	6	7	5	2	8	3	4
	**7**	0.967103657	0.963566326	0.961669232	0.962373651	0.967103657	0.956387075	0.966444468	0.965843939
		1	5	7	6	2	8	3	4
	**9**	0.968040816	0.96550586	0.962682036	0.96518338	0.968040816	0.953197992	0.96605538	0.966103255
		1	5	7	6	2	8	4	3
	**11**	0.966698706	0.964547351	0.962881108	0.965374855	0.966698706	0.95244396	0.963457452	0.96310098
		1	4	7	3	2	8	5	6
	**15**	0.962395008	0.961075951	0.958097763	0.958592014	0.962395008	0.951261012	0.957889811	0.958257796
		1	3	6	4	2	8	7	5
	**20**	0.952285735	0.950601859	0.950153839	0.945389883	0.952285735	0.949110132	0.945063572	0.946654672
		1	3	4	7	2	5	8	6
	**Average rank**	1.375	5	6.5	5.25	2.375	6.75	4.5	4.25
	**Overall rank**	1	5	7	6	2	8	4	3
**FSIM**	**2**	0.783245444	0.790174201	0.788739207	0.783965799	0.783245444	0.805333805	0.783245444	0.783245444
		5	2	3	4	6	1	7	8
	**3**	0.886654812	0.870148206	0.878722122	0.885229763	0.886654812	0.868816912	0.886599195	0.88690713
		2	7	6	5	3	8	4	1
	**5**	0.885382757	0.893019888	0.891961941	0.889100939	0.885382757	0.891789648	0.882362577	0.882370403

		5	1	2	4	6	3	8	7
	**7**	0.9012638	0.894508085	0.893920648	0.896561566	0.9012638	0.893064994	0.897005489	0.900547928
		1	6	7	5	2	8	4	3
	**9**	0.902920496	0.900038579	0.89580099	0.898609319	0.902920496	0.8817794	0.892921069	0.89589906
		1	3	6	4	2	8	7	5
	**11**	0.898404241	0.895702624	0.891232473	0.898754889	0.898404241	0.876705235	0.885735163	0.88932479
		2	4	5	1	3	8	7	6
	**15**	0.89414655	0.893541697	0.885523111	0.883811352	0.89414655	0.871885261	0.878920767	0.884187061
		1	3	4	6	2	8	7	5
	**20**	0.881693989	0.876764771	0.871016988	0.869716933	0.881693989	0.86613282	0.866757318	0.869968346
		1	3	4	6	2	8	7	5
	**Average rank**	2.25	3.625	4.625	4.375	3.25	6.5	6.375	5
	**Overall rank**	1	3	5	4	2	8	7	6

Table S10. The comparison of statistical results of the values of performance evaluation metrics for all algorithms on test image 'img10'.
